# The Role of DNA Methylation in Xylogenesis in Different Tissues of Poplar

**DOI:** 10.3389/fpls.2016.01003

**Published:** 2016-07-12

**Authors:** Qingshi Wang, Dong Ci, Tong Li, Peiwen Li, YuePeng Song, Jinhui Chen, Mingyang Quan, Daling Zhou, Deqiang Zhang

**Affiliations:** ^1^National Engineering Laboratory for Tree Breeding, College of Biological Sciences and Technology, Beijing Forestry UniversityBeijing, China; ^2^Key Laboratory of Genetics and Breeding in Forest Trees and Ornamental Plants, Ministry of Education, College of Biological Sciences and Technology, Beijing Forestry UniversityBeijing, China

**Keywords:** DNA cytosine methylation, different transcripts, methylation-sensitive amplified polymorphism (MSAP), tissue-specific DNA methylation, gene expression, poplar

## Abstract

In trees, xylem tissues play a key role in the formation of woody tissues, which have important uses for pulp and timber production; also DNA methylation plays an important part in gene regulation during xylogenesis in trees. In our study, methylation-sensitive amplified polymorphism (MSAP) analysis was used to analyze the role cytosine methylation plays in wood formation in the commercially important tree species *Populus tomentosa*. This analysis compared the methylation patterns between xylem tissues (developing xylem and mature xylem) and non-xylem tissues (cambium, shoot apex, young leaf, mature leaf, phloem, root, male catkin, and female catkin) and found 10,316 polymorphic methylation sites. MSAP identified 132 candidate genes with the same methylation patterns in xylem tissues, including seven wood-related genes. The expression of these genes differed significantly between xylem and non-xylem tissue types (*P* < 0.01). This indicated that the difference of expression of specific genes with unique methylation patterns, rather than relative methylation levels between the two tissue types plays a critical role in wood biosynthesis. However, 46.2% of candidate genes with the same methylation pattern in vascular tissues (cambium, phloem, and developing xylem) did not have distinct expression patterns in xylem and non-xylem tissue. Also, bisulfite sequencing and transcriptome sequencing of *MYB, NAC* and *FASCICLIN-LIKE AGP 13* revealed that the location of cytosine methylation in the gene might affect the expression of different transcripts from the corresponding gene. The expression of different transcripts that produce distinct proteins from a single gene might play an important role in the regulation of xylogenesis.

## Introduction

Trees constitute major parts of the biosphere and provide renewable resources for energy, pulp, paper products, and building materials (Plomion et al., 2001). *Populus tomentosa*, an important commercial tree species in northern China has been used as a model tree for biochemical analysis of lignin synthesis and for identification of candidate genes that function in lignocellulosic biosynthesis and growth, using association analysis (Du et al., 2013). However, the epigenetic mechanisms that regulate these candidate genes are just beginning to be explored. For example, DNA methylation has a crucial effect in regulating the expression of genes participating in growth, development, and disease resistance (Stroud et al., 2013). In poplar, drought stress can alter transcript levels by differentially affecting DNA methylation patterns (Raj et al., 2011). Lang-Mladeka et al. (2010) also found that DNA methylation regulated gene expression under biotic stress and abiotic stress conditions. Although, DNA methylation plays a critical role in wood formation (Plomion et al., 2001), the mechanisms by which DNA methylation affects complex traits controlling wood synthesis and xylogenesis and the tissue-specific variation in DNA methylation remain relatively unexplored.

Previous research suggested that different DNA methylation levels and patterns exist in different species (Lira-Medeiros et al., 2010). Moreover, DNA methylation also varies with age and substantial evidence in several species has shown tissue-specific variation in DNA methylation. Zhang et al. (2006) investigated the difference in absolute methylation in multiple tissues of *Arabidopsis thaliana* and pair-wise comparisons showed significant differences among these tissues. In annual plant species, although few studies have compared methylation profiles among tissues, work in Arabidopsis showed that cytosines in the CG, CHG, and CHH contexts (Cokus et al., 2008) were differentially methylated in different tissues (Lister et al., 2008). Work in rice showed similar results, as patterns of 5 mC in specific sites differed between leaves and roots and affected transcription of neighboring genes (Kashkush and Khasdan, 2007). Vining et al. (2012) reported that DNA methylation differences were evident among tissues from *P. trichocarpa*; however, they examined relative few types of tissue. Furthermore, relatively little is known about the distribution of DNA methylation within different tissues and the variety of DNA methylation among different tissues in plants, especially in perennial species such as trees.

Forest trees undergo long-term tissue differentiation and have long generation times; moreover, their perennial nature means they must acclimate to changing environments. Previous studies on the genetic diversity and population structure of *P. tomentosa* have provided an excellent theoretical foundation for further research and breeding work (Du et al., 2012). However, the phenotypic variation is not only decided by the DNA sequence, as evidence suggests a strong dependence on epigenetic modifications, especially DNA methylation (Cubas et al., 1999; Jones and Takai, 2001).

With the development of new techniques, DNA methylation can be studied through a variety of methods, such as bisulfite sequencing (BS-Seq), methylated DNA immunoprecipitation (MeDIP), methylation-sensitive amplified polymorphism (MSAP), and others. Among these methods, MSAP is a stable, and high-throughput method to investigate cytosine methylation at the genomic level without *a priori* knowledge of the genomic sequence (Cervera et al., 2002; Verhoeven et al., 2010).

Here, we studied xylogenesis by investigating the role that tissue-specific DNA methylation plays in regulating the expression of wood-related genes. We examined the DNA cytosine methylation of 10 tissues and organs from *P. tomentosa* by using MSAP markers and the correlation between gene expression and DNA cytosine methylation. MSAP markers were sequenced and analyzed to obtain candidate genes, which were verified by BS-seq. Moreover, some genes produced different transcripts in response to changes in DNA methylation; we predicted the protein structure and function of the products of these different transcripts to examine the effect of epigenetics on these loci. This work provides reference data for the study of the effect of methylation on wood formation and regulating gene expression in *Populus* and other trees.

## Materials and methods

### Plant materials

Ten tissues and organs were collected from the *P. tomentosa* clone “LM50” cultivated in the national nursery of Guan Xian County, Shandong Province, China (36⋅23′N, 115⋅47′E) were used in our study. Shoot apex, young leaf, mature leaf, phloem, cambium, developing xylem, mature xylem, and root were collected from a 1-year-old tree at 10:00 a.m. on June 10th 2012. Male and female catkins from two same-age flowering individuals were collected at the last phase of flower development, before pollination. Three biological replicates were used for each tissue and organ. All the samples were immediately frozen in liquid nitrogen, and then stored at −80⋅C until they were used for DNA and RNA extraction.

### Genomic DNA and RNA extraction

Plant samples were ground to powder with liquid nitrogen and genomic DNA was isolated using the DNeasy Plant Mini kit (Qiagen China, Shanghai). Total RNA from mature leaf, phloem, cambium, developing xylem, and mature xylem was isolated using the RNA isolation kit (Autolab Biotechnology, Beijing, China). Additional on-column DNase digestions were performed three times during the RNA purification using the RNase-Free DNase Set (Qiagen). The quantity and quality of extracted DNA and RNA were detected with a NanoDrop ND-1000 (Thermo, USA), and quantified RNA was reverse transcribed into cDNA using the SuperScript First-Strand Synthesis system and the supplied polythymine primers (Invitrogen).

### Methylation-sensitive amplified polymorphism (MSAP) analysis

MSAP is modified from the Amplified Fragment Length Polymorphism (AFLP) approach (Vos et al., 1995), in which *Mse*I is replaced by isoschizomeric endonucleases *Hpa*II or *Msp*I as frequent cutters in parallel reactions. These two endonucleases show differential sensitivity to the cytosine methylation state of their recognition site 5′-CCGG (McClelland et al., 1994): both *Hpa*II and *Msp*I cut the unmethylated 5′-CCGG, but *Hpa*II does not cut if either of the cytosines is double-strand methylated, whereas *Msp*I does not cut if the external cytosine is hemi-methylated (Dong et al., 2006; Figure S1). Since either methylated cytosine at the 5′-CCGG recognition site is the sensitive residue of *Hpa*II, and *Msp*I is sensitive just to the external methylated cytosine, the internal methylated cytosine would lead the bands to appear in the pattern 0, 1, where 0 represents the absence of the band and 1 represents the presence of the band (Figure S2) in the denatured polyacrylamide and capillary electrophoresis sequencing gels loaded with the selective amplification products of *Eco*RI/*Hpa*II and *Eco*RI/*Msp*I, respectively. Meanwhile, a few fragments are present among the selective amplification products of the *Eco*RI/*Hpa*II digest but not in the *Eco*RI/*Msp*I (pattern 1, 0; Figure S1). The non-methylated sites have the pattern 1, 1 (Figure S1). The detailed MSAP protocol was described by Zhong et al. (2009) and Ma et al. (2012). One hundred and thirty-five selective primer combinations (Table S1) were used to detect the cytosine methylation of 10 tissues and organs in our study and each primer contained three additional selective nucleotides at the 3′ ends. All adapters and primers (Table S2) were synthesized in Tsingke (Beijing, China), the HM selective primers were 5′-fluorescent labeled with 6-carboxyfluorescein.

### Sequencing of polymorphic MSAP fragments

The polymorphic MSAP fragments for 10 tissues and organs were excised from the denatured polyacrylamide gel and dissolved in 80 μl of deionized water in PCR tubes, then incubated at 95⋅C for 10 min. A 3-μl sample was used as a template to re-amplify with the same primer combinations under the same amplification conditions used for the selective amplification. The fresh DNA products of re-amplification were recovered using High Yield Nucleic Acid Purification Kit for Small DNA Fragments (BioDev-Tech, Beijing, China) and cloned into the *pEASY*-T1 Simple Cloning Vector (TransGen Biotech, Beijing, China). *E. coli* strain TOP10 (Biomed, Beijing, China) was transformed for sequencing. The sequences obtained were analyzed using NCBI BLAST algorithms (http://blast.ncbi.nlm.nih.gov/Blast.cgi), and PopGenIE (http://popgenie.org/), which provided the gene's structure, including promoter, 5′ UTR, exons, introns, and 3′ UTR. Candidate genes related to wood formation were obtained by searching a database of wood formation-related genes (http://me.lzu.edu.cn/woodformation/index.php).

### Bisulfite sequencing of candidate genes

For analysis of the candidate gene related to wood formation, genomic DNA was treated with bisulfite and used as template for amplification, which was carried out for 35 cycles, with primers designed using Methyl Primer Express (v1.0) software (Herman et al., 1996). Each candidate gene was amplified using multiple primers to amplify the whole gene sequence. Primers are listed in Table S3. PCR products were then purified using a Gel Extraction Kit (Qiagen, Hilden, Germany) and cloned into the vector pMD18-T (Takara Bio, Inc., Tokyo, Japan). Fifteen positive clones for each individual were selected for sequencing through an ABI sequencer (PRISM BigDye Terminator, ABI, Sunnyvale, CA, USA). All sequencing was performed on the three replicates.

### Methylation specificity calculation for methylated genes

To measure tissue- or organ-specificity of methylation of genes that the sequenced MSAP fragments matched, the tau score was computed by defining the methylated sites as “1” and the non-methylated and uninformative sites as “0.” Let *M*_*ij*_ be the defined numerical value of gene *i* in tissue *j*. Then the tissue specificity of gene *i* is given by:

$$\tau \text{​}i=\frac{1}{n-1}\left(\sum_{j\;=\;1}^{n}Mij-1\right)$$

where *n* is the number of tissues. Thus, if the methylation pattern of a gene is the same in all tissues and organs the tau score is one, and if a gene is methylated in only one tissue or organ the tau score is zero.

### Real-time quantitative reverse-transcription PCR (qRT-PCR)

The cDNAs synthesized from mRNAs of mature leaf, phloem, cambium, developing xylem, and mature xylem were diluted and used to perform qRT-PCR with the DNA Engine Opticon 2 machine (MJ Research, USA). The mixture contained 2 μl cDNA, 0.5 μl each of the primer (10 mM), 12.5 μl SYBR (Cat. RR420A, TaKaRa), and 9.5 μl ddH_2_O. The PCR protocol initiated with a denaturation at 94⋅C for 5 min, then 40 cycles of 30 s at 94⋅C, 30 s at 58⋅C, and 30 s at 72⋅C, and a final melt-curve 70–95⋅C. The melting curve was used to check the specificity of the amplified fragments (Zhang et al., 2011). All reactions were carried out in triplicate for technical and three individuals for biological repetitions, respectively, and the generated real-time data were analyzed using the Opticon Monitor Analysis Software 3.1 tool. Primer Express 3.0 software (Applied Biosystems) was used to design the specific primer pairs for target genes (Table S4). Poplar *Actin* (Accession Number: EF145577) was used as the internal control. One hundred and thirty-two differentially methylated genes (Table S4) among 10 different tissues and organs were chosen for qRT-PCR verification.

### Prediction of protein structure and function

The whole gene and protein sequences were obtained from the genome database of poplar, PopGenIE (http://popgenie.org/). Moreover, the annotated protein function was acquired from ExPASy (http://www.expasy.org/) and the prediction of protein structure was obtained from SIB (http://swissmodel.expasy.org/interactive).

### Statistical analysis

The data on methylation patterns were fitted to the SPSS 20 (Ahlrichs et al., 1989), and a significance threshold of *P* < 0.05 or *P* < 0.01 was used in general. Means of different relative methylation pattern levels within individual tissues were compared using a *t*-test. Significant correlations were calculated using Pearson's *r* coefficient and examined by a *t*-test.

## Results

### Global methylation patterns in different tissues and organs from *Populus*

**Figure 1:**
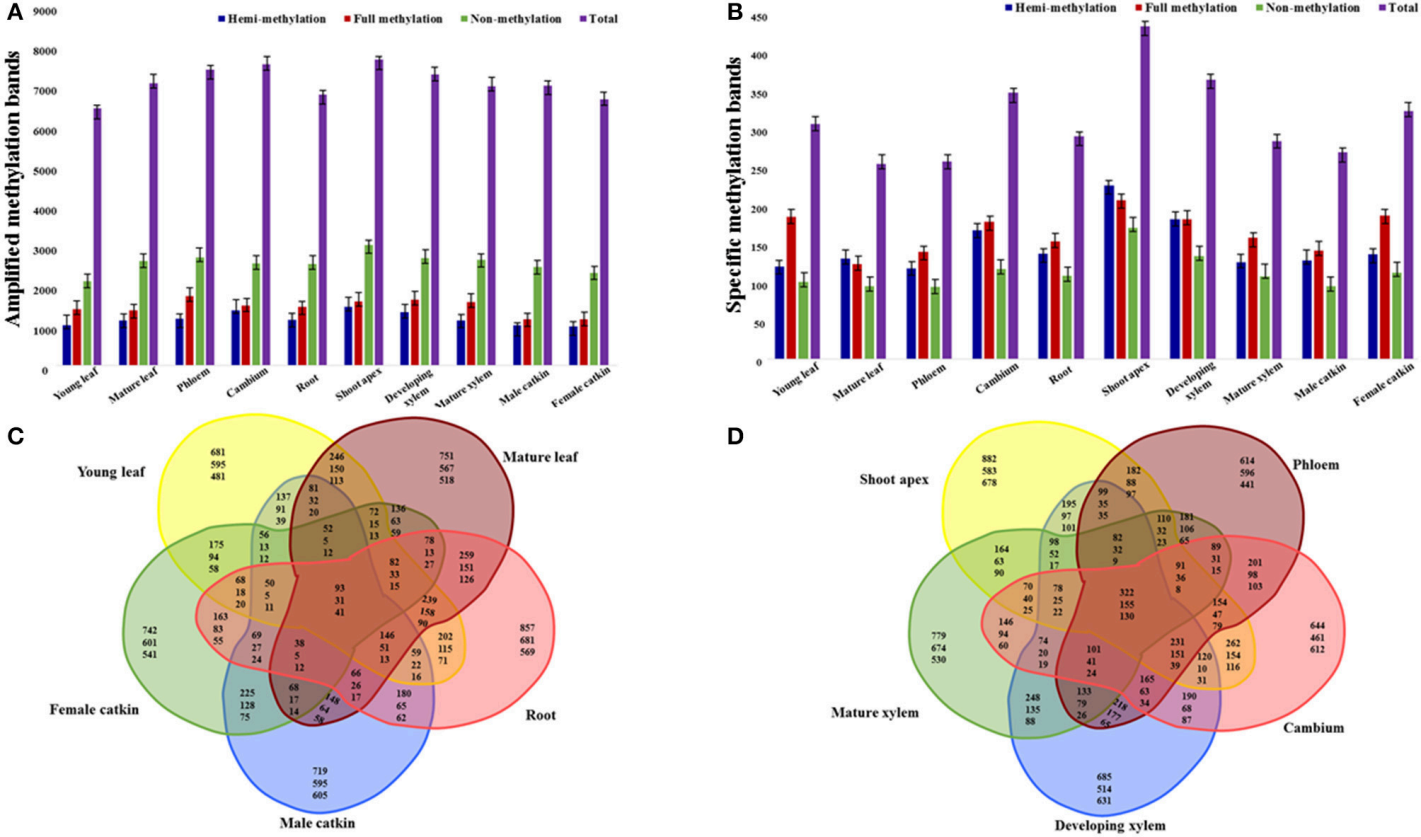
**Amplification of selective amplification primer combinations in tissues and organs of *P. tomentosa*.** Numbers of amplified methylation bands **(A)** and specific methylation bands **(B)** detected in each tissue or organ type are shown in colored columns, which represent hemi-methylated, fully methylated, non-methylated, and total methylated bands from left to right. **(C)** Venn diagram illustrating shared and unique methylated sites (top numbers), fully methylated sites (middle numbers), and hemi-methylated sites (bottom numbers) among the five organ types. **(D)** Venn diagram illustrating shared and unique methylated sites (top numbers), fully methylated sites (middle numbers), and hemi-methylated sites (bottom numbers) among the five tissue types.

To study the cytosine methylation profiles of different tissues and organs, we used MSAP to measure methylation levels across the genome in *P. tomentosa*. The 135 primer combinations amplified 11–204 bands of 55–550 bp, with an average of 93 bands per primer-pair. In total, this amplified 12,575 bands, of which 10,316 (82.0%) were polymorphic (Table S1). The average number of polymorphic loci for the primer combinations was 76, with E40+H/M46 generating the fewest (11) and E65+H/M33 generated the most polymorphic (185) bands (Table S1). Different types of tissues and organs generated different patterns of amplification bands, with shoot apex showing the most bands (7838), and young leaf showing the fewest (6577) (Figure 1A). Male catkins had the most non-methylated sites (4946), and young leaf had the fewest (4112) (Figure 1A). Shoot apex (hemi-methylated 1500) and phloem (fully methylated 1780) had the most hemi-methylated and fully methylated sites, and female catkin had the fewest (990 and 1177, respectively; Figure 1A). In general, we observed more methylated sites in the shoot apex than in other samples, showing more frequent methylation in this tissue.

**Figure 2:**
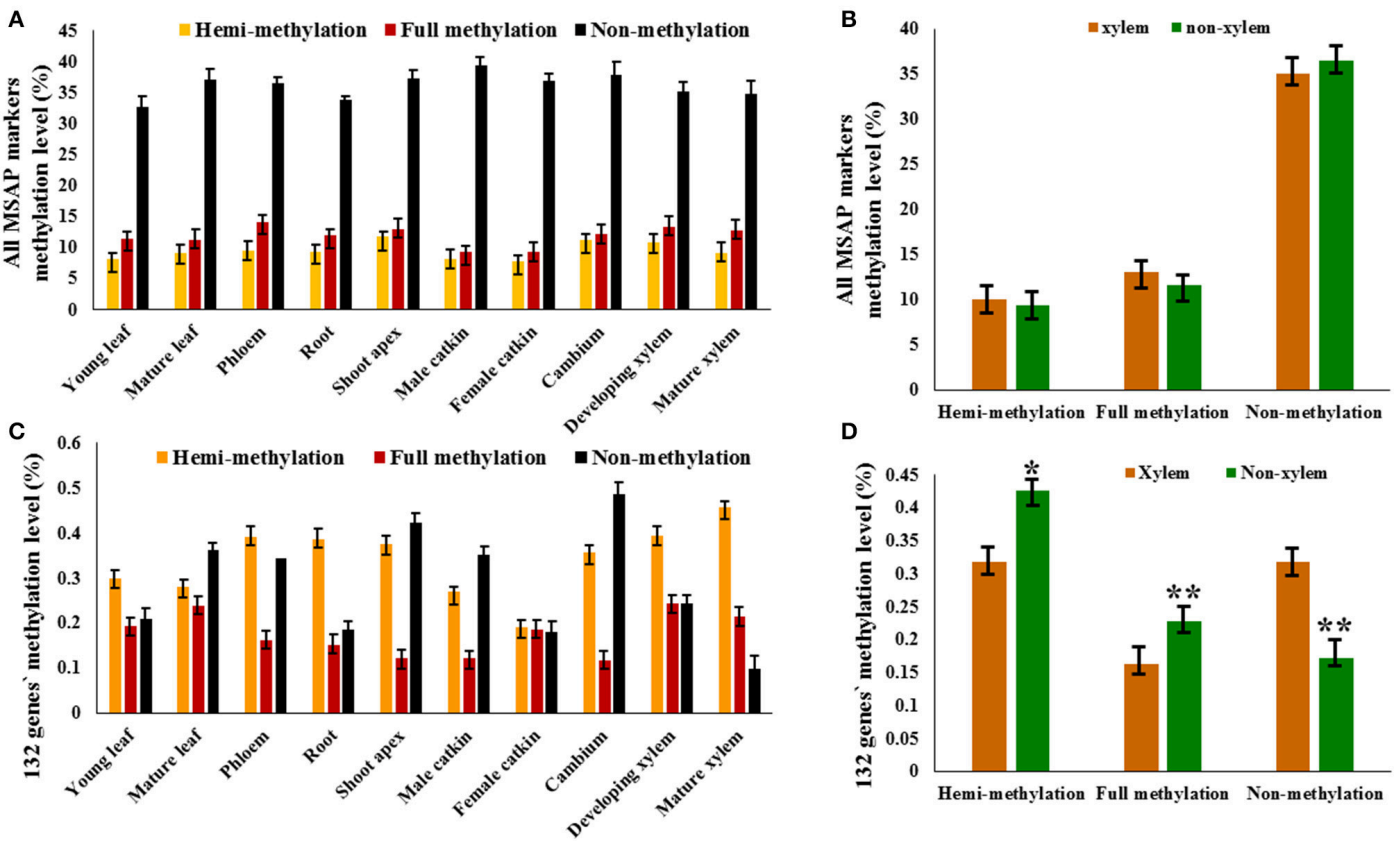
**MSAP markers and 132 candidate genes in tissues and organs of *P. tomentosa*.** The relative methylation level of MSAP marker **(A)** and candidate genes **(C)** detected in each tissue or organ type are shown in colored columns, which represent hemi-methylated, fully methylated, and non-methylated from left to right. **(B)** The difference between xylem tissues and non-xylem tissues exists in the relative hemi-methylation level (*P* = 0.628), the relative full methylation level (*P* = 0.254), and the relative non-methylation level (*P* = 0.389). **(D)** The significantly difference between xylem tissues and non-xylem tissues exists in the relative hemi-methylation level (*P* = 0.043), the relative full methylation level (*P* = 0.003), and the relative non-methylation level (*P* = 0.001). *P < 0.05; **P < 0.01.

We defined the non-methylation and methylation levels in each DNA sample as percentages of the different patterns' marker amounts and the total markers, which were equal to the total number of bands. Therefore, because the detected methyl-cytosines were located at 5′-CCGG sites and uninformative methylation status could not be clearly determined, the methyl-cytosine level obtained was relative. The relative non-methylation levels (average 36.17%) of all tissues were more than the relative methylation levels (average 10.71%), and relative full methylation levels (average 11.90%) were more than the relative hemi-methylation levels (average 9.52%; Figure 2A). The relative full methylation level ranged from 9.36% in the female catkin to 14.16% in the phloem with the young leaf, mature leaf, male catkin, and female catkin were less than the average level, and the relative hemi-methylation level ranged from 7.87% in the female catkin to 11.93% in the shoot apex with the cambium, shoot apex, and developing xylem having greater than average levels (Figure 2A). Thus, MSAP analysis indicated that cytosine methylation for CpG-sites differed substantially among tissues and organs, with approximate 85% of the detected loci showing differential methylation and only 0.34% of the methylated loci showing common patterns of methylation in the different tissues and organs. Other sites were non-methylated or uninformative.

### Tissue or organ specific methylation and dynamic of methylation sites

Specific cytosine methylation of tissues and organs at CpG-sites was calculated and of all the 12,575 loci, 3371 (26.81%) had tissue- or organ-specific cytosine methylation (Figure 1B). The shoot apex had the most specific hemi- (244), full (223), and total (467) cytosine methylation sites. The phloem had the fewest specific hemi-methylation sites (127), and the mature leaf had the fewest full and total methylation sites (133 and 274 sites, respectively; Figure 1B). All five organs (i.e., young leaf, mature leaf, root, male catkin, and female catkin) had 93 shared sites, including 31 fully methylated and 41 hemi-methylated sites (Figure 1C). The young leaf, mature leaf, root, and male catkin shared the most methylated sites (146), while mature leaf, root, male catkin, and female catkin shared the fewest sites (38) (Figure 1C). The shoot apex, phloem, cambium, developing xylem, and mature xylem shared 322 sites and the shoot apex, cambium, developing xylem, and mature xylem shared 78 sites (Figure 1D). More shared fully methylated sites (155) and shared hemi-methylated sites (130) of five tissues than five organs were detected (Figure 1D).

Furthermore, dynamic of methylation sites in different developmental stages was detected. One thousand four hundred and fifty-four sites were methylated in young leaf but non-methylated in mature leaf, 139 fully methylated sites of young leaf changed to hemi-methylated in mature leaf, and 72 hemi-methylated sites in young leaf changed to full methylation in mature leaf (Table 1). Also in the vascular tissues, 1216 sites were methylated in cambium but were non-methylated in both phloem and mature xylem, and 368 methylated sites in cambium were non-methylated in phloem but were still methylated in mature xylem. Seven hundred and fifty-one methylated sites in cambium were methylated in phloem but changed to non-methylated in mature xylem. Thirty-four fully methylated sites in cambium changed to hemi-methylation in phloem but remained fully methylated in mature xylem, and 32 fully methylated sites in cambium were still fully methylated in phloem but changed to hemi-methylation in mature xylem. Twenty-eight cambium hemi-methylated sites changed to full methylation in phloem but were still hemi-methylated in mature xylem and 33 cambium hemi-methylated sites were still hemi-methylated in phloem but changed to fully methylated in mature xylem (Table 1). These results revealed dramatic alterations of DNA methylation patterns among different tissues and organs and related to developmental stage.

**Table 1:** **Dynamic of cytosine methylation patterns in different development stages of *Populus tomentosa***.

**Tissue**	**Young leaf**	**Mature leaf**	**Phloem**	**Cambium**	**Mature xylem**	**Number of site**
Methylation pattern	M	N				1454
	F	H				139
	H	F				72
			N	M	N	1216
			N	M	M	368
			M	M	N	751
			H	F	F	34
			F	F	H	32
			F	H	H	28
			H	H	F	33

*F, H, M, and N represented full methylation, hemi-methylation, methylation, and non-methylation pattern, respectively*.

### Sequence analysis of polymorphic MSAP fragments

To examine the genomic sequence surrounding the methylated sites, we excised 241 polymorphic MSAP fragments (*MF*) (from 64 to 549 bp in length) from the gel and re-amplified them to obtain their sequences. From these sequences, we used NCBI BLAST (http://blast.ncbi.nlm.nih.gov/Blast.cgi) and PopGenIE (http://popgenie.org/) to map 210 genes. Except for 20 MSAP fragments without similarity to known proteins, the remaining 190 genes encode proteins that showed sequence similarity to known proteins.

Classified by molecular function, 15 genes (*MF12, MF17, MF56, MF67, MF72, MF93, MF97, MF100, MF136, MF145, MF161, MF172, MF191, MF197*, and *MF198*) encode putative kinases. For example, MAPK/ERK kinase kinase 1 (*MF12*) acts in a pivotal signaling pathway that can be activated by multiple extracellular stimuli and transmits signals to diverse substrates. *MF56* encodes HERCULES1, a receptor kinase regulated by brassinosteroids and required for cell elongation during vegetative growth (Table 2, Table S5). The mapped genes also included translation factor genes, encoding proteins such as translation initiation factor SUI1 (*MF70*), eukaryotic translation initiation factor 2 gamma subunit (*MF71*), and enhancer of polycomb-like transcription factor protein (*MF157*). *MF32, MF84*, and *MF95* matched genes encoding the DNA-binding protein HORMA, WRKY DNA-binding protein 9, and basic helix-loop-helix (bHLH) DNA-binding superfamily protein. *MF121* and *MF202* matched genes encoding an RNA-binding (RRM/RBD/RNP motifs) family protein and RNA-binding KH domain-containing protein. Molecular chaperone genes, encoding proteins such as an HSP20-like chaperone family protein (*MF62*), heat shock protein DnaJ with tetratricopeptide repeat (*MF115*), and chaperone DnaJ-domain superfamily protein (*MF135*), were also mapped. Furthermore, methylation-related genes (encoding O-methyltransferase 1 and protein arginine methyltransferase 4A) were identified (Table S5). All the above methylated genes participate in various biological processes, which should undergo different regulation during different developmental and environmental responses.

**Table 2:** **Characterization of the polymorphic MSAP fragments that mapped to genes related to wood properties**.

**Fragment**	**Length (bp)**	**Reference gene ID**	**Methylation location**	**Protein annotation**
MF1	160	Potri.014G155200	Exon	Alpha/beta-hydrolases superfamily protein
MF2	315	Potri.014G008900	3′ flanking region	Heptahelical protein 4
MF17	125	Potri.006G051700	Exon	Leucine-rich repeat protein kinase family protein
MF21	100	Potri.001G258700	Exon	Myb domain protein 46
MF36	319	Potri.009G133400	Exon	Subtilisin-like serine protease 2
MF44	132	Potri.018G008900	Exon	UDP-glycosyltransferase 73B4
MF96	169	Potri.011G093400	Exon	Pectin lyase-like superfamily protein
MF98	130	Potri.017G036700	Exon	Raffinose synthase family protein
MF117	143	Potri.003G077900	Exon	Plant protein of unknown function (DUF828) with plant pleckstrin homology-like region
MF147	138	Potri.006G129200	Exon	FASCICLIN-like arabinogalactan-protein 11
MF148	222	Potri.005G228400	Exon	Oxidoreductases, acting on NADH or NADPH
MF161	123	Potri.001G353100	Exon	Kinase-related protein of unknown function (DUF1296)
MF171	292	Potri.006G255000	5′ UTR	ENTH domain-containing protein/clathrin assembly phospholipid binding protein
MF173	424	Potri.003G201000	Promoter	Senescence-associated E3 ubiquitin ligase 1
MF174	177	Potri.001G024600	Intron	ARM repeat superfamily protein
MF175	139	Potri.002G172400	Exon	Cellular apoptosis susceptibility protein, putative/importin-alpha re-exporter, putative
MF176	260	Potri.014G100000	Exon	Cellular apoptosis susceptibility protein, putative/importin-alpha re-exporter, putative
MF197	139	Potri.008G027000	Exon	Aspartate/glutamate/uridylate kinase family protein
MF201	377	Potri.006G158700	Exon	Polyol/monosaccharide transporter 5
MF204	435	Potri.017G092000	Exon	UDP-glucose 6-dehydrogenase family protein
MF205	221	Potri.014G037900	Exon	Cytochrome P450, family 82, subfamily C, polypeptide 4
MF206	142	Potri.007G117800	Exon	Exostosin family protein
MF218	165	Potri.017G086100	Exon	Pentatricopeptide repeat (PPR) superfamily protein

Among the 210 methylated genes, the methylation patterns of 93 genes showed very strong differences among tissues and organs. For example, Potri.003G057100 (*MF18*) was hemi-methylated in mature leaf and developing xylem, was fully methylated in cambium, shoot apex, mature xylem, and female catkin, but was non-methylated in phloem and male catkin (Table S6). To measure the tissue- or organ-specificity of methylation of these genes, we computed the tau score, where a tau of one indicates low tissue or organ specificity and a tau of zero indicates high specificity (see Materials and Methods). Potri.009G041200 (*MF58*) and Potri.002G156200 (*MF233*) got a tau score of 1, indicating that they tended to be uniformly methylated in different tissue and organ types. Eleven genes had a tau score of 0, indicating tissue- or organ-specific cytosine methylation; for example Potri.008G179700 (*MF33*), Potri.017G078100 (*MF132*), and Potri.004G177700 (*MF151*) had female catkin specific methylation. Also, Potri.005G251100 (*MF114*), and Potri.019G010000 (*MF135*) were fully methylated specifically in mature xylem and developing xylem, respectively (Table S6).

### The difference between xylem tissue and non-xylem tissue

To analyze the role DNA methylation plays in wood formation, 10 tissues were classified into two tissue types, xylem tissue (developing xylem and mature xylem) and non-xylem tissue (cambium, shoot apex, young leaf, mature leaf, phloem, root, male catkin, and female catkin), based on xylogenesis. Relative DNA methylation level was determined by calculating the pattern of 10,316 methylation polymorphic sites obtained by capillary electrophoresis in the 10 tissues. The relative hemi-methylation level was 7.87–11.93%; the relative full methylation level was 9.36–14.16%; the relative non-methylation level was 32.70–39.33% (Figure 2B). However, these did not significantly differ between xylem and non-xylem tissue types, with a P value of 0.628 for the difference in relative hemi-methylation level between xylem and non-xylem tissue, 0.254 for the relative full methylation level, and 0.389 for the relative non-methylation level (Figure 2B).

However, the relative methylation levels of 132 genes (selected from 210 MSAP markers purified by denaturing polyacrylamide gel electrophoresis), which have the same methylation patterns in developing xylem and mature xylem, did significantly differ between xylem tissues and non-xylem tissues (P values of 0.043 for hemi-methylation, 0.003 for full methylation, and 0.001 for non-methylation; Figures 2C,D). This indicated that the methylation pattern of specific genes, rather than the methylation level of the whole genome, might have a potential relationship with xylogenesis.

Seven candidate genes closely related to wood development were identified using WFRGD (http://me.lzu.edu.cn/woodformation/index.php). *MF42, MF69, MF84, MF147, MF200, MF204*, and *MF224* matched wood formation-associated genes, like those encoding a MYB-like HTH transcriptional regulator, NAC (No Apical Meristem) domain transcriptional regulator superfamily protein, WRKY DNA-binding protein 9, FASCICLIN-like arabinogalactan-protein 11, Basic helix-loop-helix (bHLH) DNA-binding superfamily protein, UDP-glucose 6-dehydrogenase, and Laccase 17 (Table 2, Table S5).

### Correlation of methylation and gene expression

**Figure 3:**
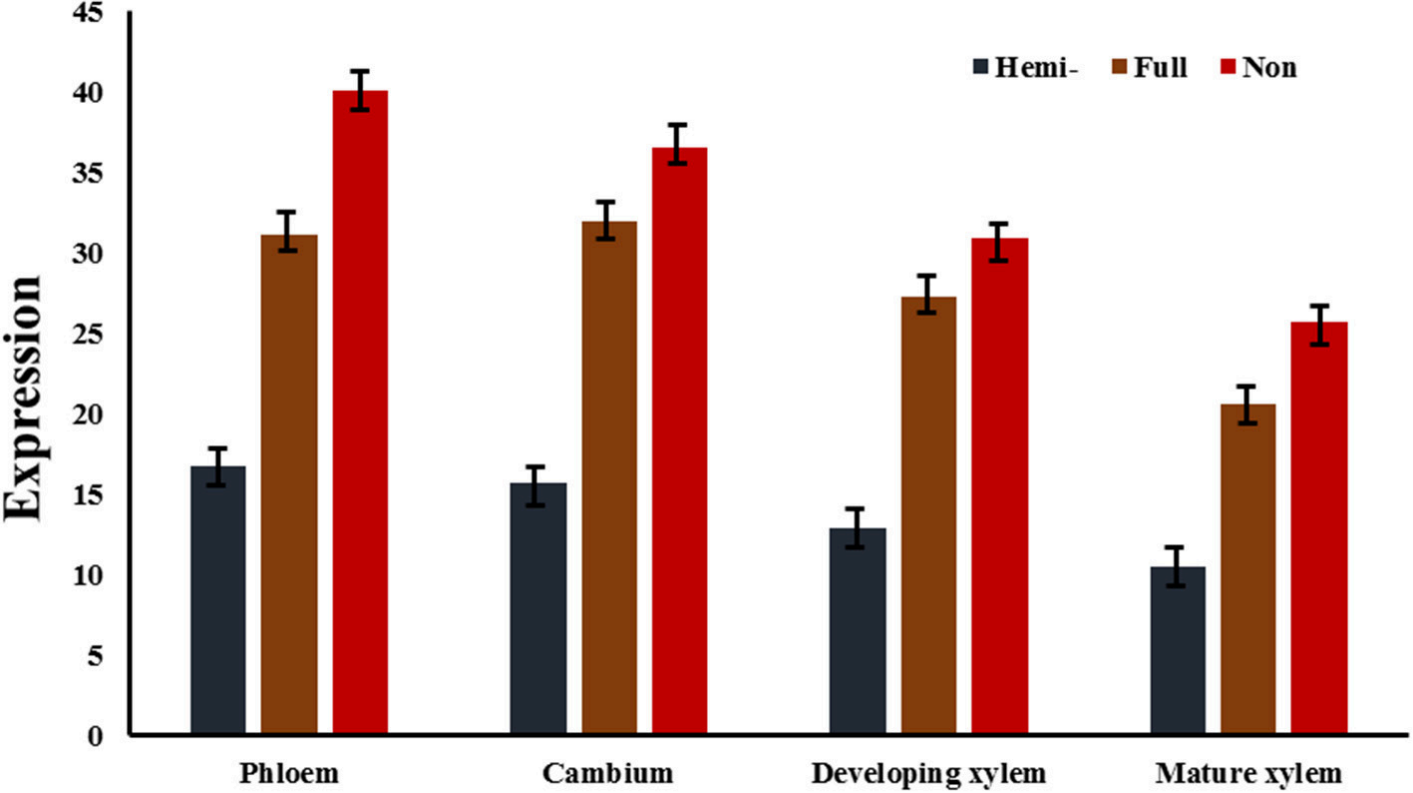
**Average expression of genes of three different methylation types in three vascular tissues**. In cambium, the differences of transcript levels of genes of three different methylation types were significant with *P*-values of 0.011 (hemi- vs. full), 0.005 (hemi- vs. non-), and 0.044 (full vs. non-), respectively. In developing xylem, the *P*-values were 0.039 (hemi- vs. full), 0.026 (hemi- vs. non-), and 0.048 (full vs. non-), respectively. In mature xylem, the *P*-values were 0.029 (hemi- vs. full), 0.009 (hemi- vs. non-), and 0.045 (full vs. non-), respectively. Hemi-, full, and non- represented average expressions of hemi-methylated genes, fully methylated genes, and non-methylated genes in three vascular tissues.

To test whether the transcript levels of genes from MSAP fragments were affected by their DNA methylation status, data on the expression of the 132 differentially methylated genes in 10 tissues were obtained by qRT-PCR (primers are listed in Table S4), except for vascular tissues (phloem, cambium, developing xylem, and mature xylem) extracted from an RNA-sequencing data set (unpublished data). The relative hemi-methylation levels were 17.42–53.79%, with cambium having the lowest level and xylem the highest level. The relative full methylation levels were 9.09–25.00%, with female catkin having the lowest level and xylem the highest level. The relative non-methylation level was lowest (9.84%) in xylem and highest in cambium (43.18%). Also, genes with hemi-methylation made up 41.14% of the total genes and fully methylated genes made up 16.36% of the total (Table 3). Calculation of Pearson's *r* coefficient suggested that gene expression is not correlated with tissue-specific variation (*r* = 0.028, *P* = 0.584), but it is related to methylation pattern (*r* = 0.574, *P* = 0.008), where for the calculation of correlation we only used four vascular tissues because we had too few valid values to compare in 10 tissues. Transcript levels of differentially methylated genes showed the same pattern of difference in the 10 tissues. In cambium, 26 genes were hemi-methylated and the transcript levels of these genes were significantly (*P* < 0.05) lower than those of the 35 fully methylated and 71 non-methylated genes (Figure 3). The transcript levels of fully methylated and non-methylated genes in cambium also significantly differed, with a *P*-value of 0.044 (Figure 3). In developing xylem and mature xylem, transcript levels of genes with different methylation patterns showed the same significant (*P* < 0.05) difference (Figure 3). DNA methylation tended to suppress gene transcription and hemi-methylation showed higher power than full methylation.

**Table 3:** **The relative methylation level of different pattern in 10 tissues**.

	**Hemi-methylated level (%)**	**Full methylation level (%)**	**Non-methylated level (%)**
Young leaf	16.67	37.88	18.94
Mature leaf	18.18	32.58	39.39
Root	12.88	50.76	16.67
Shoot apex	10.61	44.70	35.61
Male catkin	12.88	28.79	31.06
Female catkin	18.94	17.42	18.94
Phloem	14.39	48.48	28.03
Cambium	9.09	43.18	43.18
Developing xylem	25.00	53.79	9.85
Mature xylem	25.00	53.79	9.85

### The role of candidate genes in xylogenesis in vascular tissues

**Figure 4:**
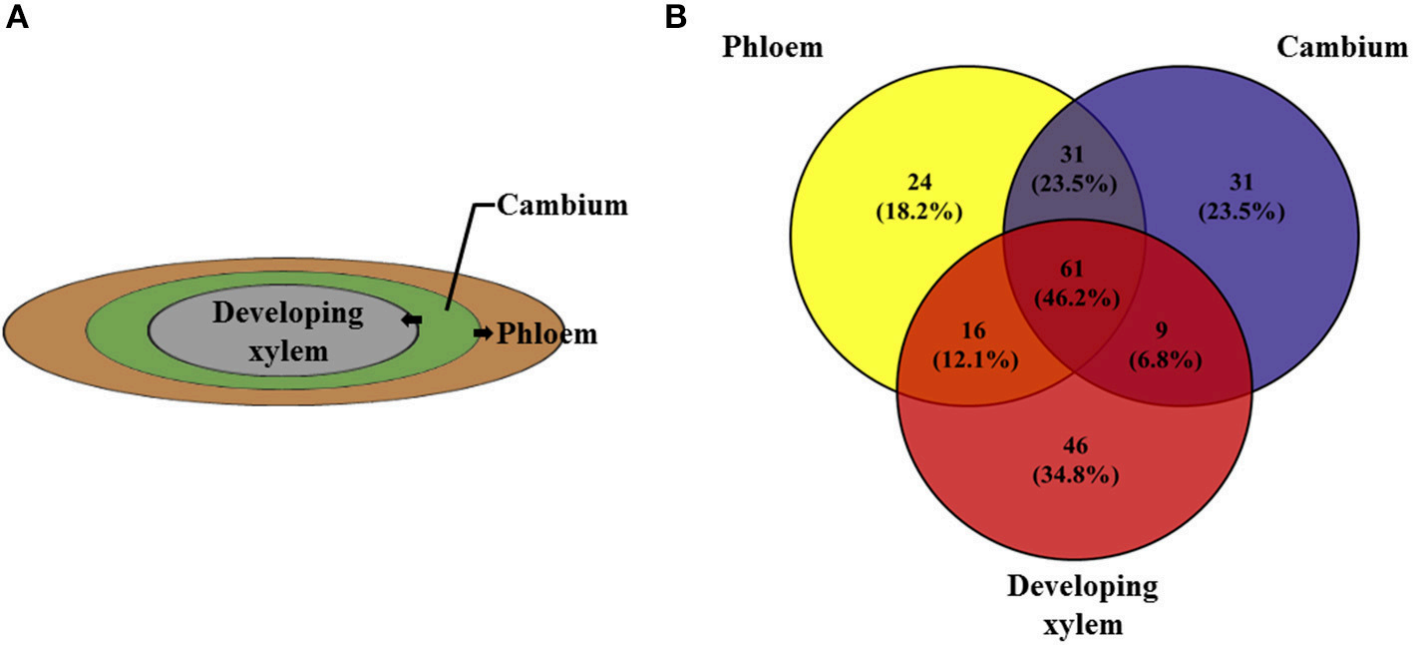
**The three important xylogenesis tissues, phloem, cambium, and developing xylem. (A)** The cells of cambium differentiate into phloem and developing xylem. **(B)** Venn diagram shows the number of 132 candidate genes of phloem, cambium and developing xylem with 61 genes having the same methylation pattern.

Gene expression did not significantly differ among the four vascular tissue types (phloem, cambium, developing xylem, and mature xylem; Figure 3), with the four tissues having 61 (46.2%) the same methylation pattern in the candidate genes (Figure 4). This verified the regulatory effect of DNA methylation on gene expression, but it also raised the question that if there is no alternative expression of genes, how can these tissues be differentiated? Wood formation is directly related to the selective transformation of cambium into phloem and developing xylem, which eventually differentiates into mature xylem. So we used phloem, cambium and developing xylem for the next analysis. Three wood-related genes, *MYB, NAC*, and *FASCICLIN-LIKE AGP 13*, were chosen to be treated with bisulfite and sequenced with multiple primer pair combinations, which showed that although there was the same methylation pattern in 5′-CCGG, actually, it differed from the methylation pattern in the whole gene (Excel S1).

**Figure 5:**
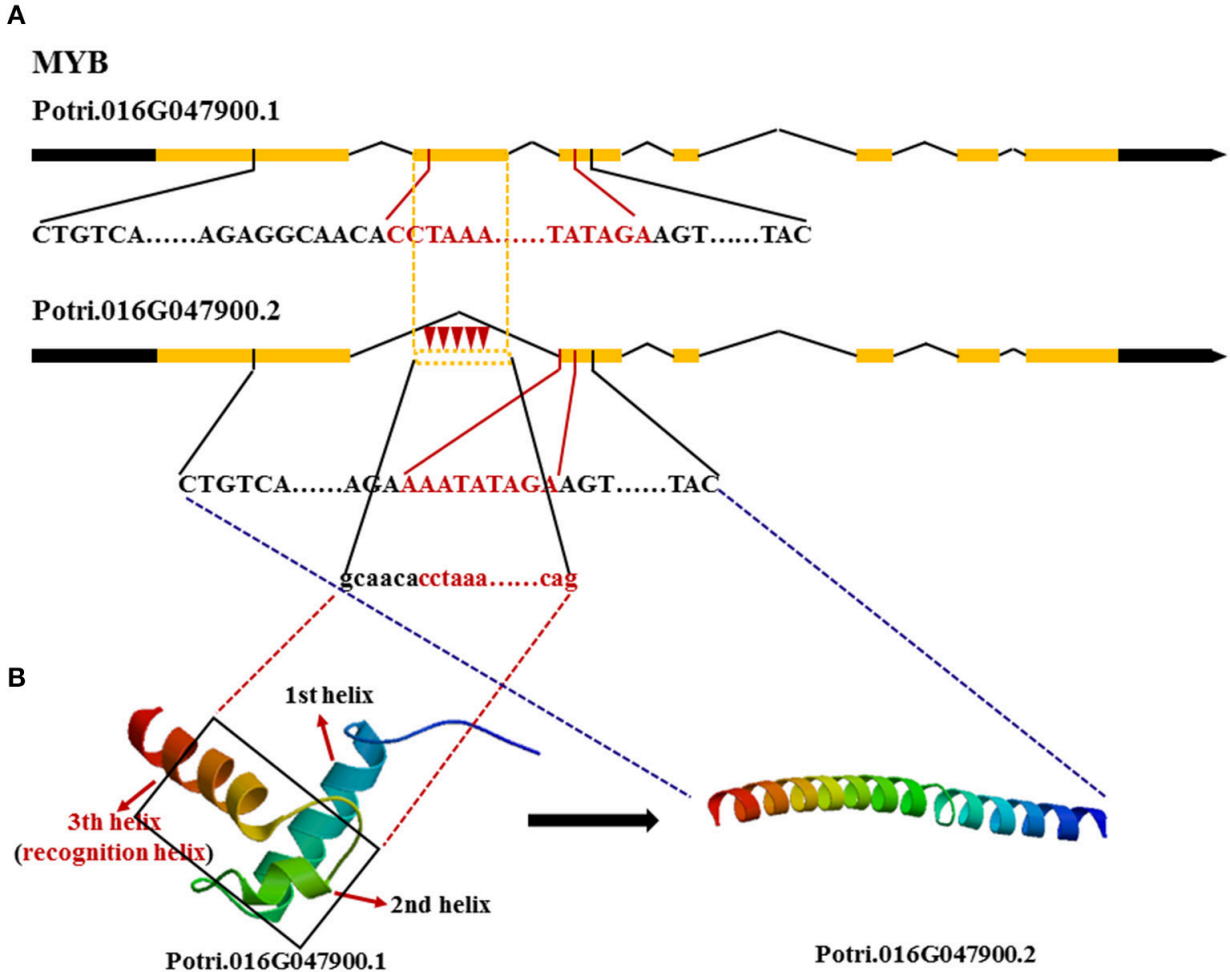
**Different transcripts of *MYB* encoding different proteins. (A) 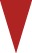
** Red inverted triangle, represents methyl and black words represent DNA encoding the functional domain where red words represent DNA encoding the DNA binding domain. **(B)** The two different 3D-structures of MYB are translated by different transcripts, respectively., 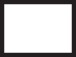 black rectangle, represents the lost part of the second exon in the second transcript.

We also tested whether these genes made different transcripts, which can be produced by alternative splicing. Because *FASCICLIN-LIKE AGP 13* has one large exon, using RNA-seq, even if different transcripts occur with a gap in the sequence, they will be mapped to the same transcript. Here, *MYB* and *NAC* were mainly analyzed using a combination of methylation PCR and RNA-seq. Two transcripts of *MYB* were obtained in the three vascular tissues; the first transcript contained all exons, while the second transcript lacked the second exon (Figure 5). It is notable that the cytosine methylation cluster occurs in the second exon in *MYB* in developing xylem with the second transcript present at higher levels (FPKM = 88.92 ± 1.18), while methylation does not tend to occur in the second exon in phloem (FPKM = 142.74 ± 5.00) and cambium (FPKM = 102.70 ± 3.29) where the first transcript is mainly expressed. *NAC* produces four transcripts. The first transcript is major component of phloem (FPKM = 220.87 ± 1.07) and cambium (FPKM = 228.27 ± 4.11) transcriptomes, but the second transcript showed highest expression in developing xylem (FPKM = 250.22 ± 8.25). The FPKM value of the third transcript was 0.81 ± 0.03 in phloem, 7.96 ± 0.27 in cambium, and 2.15 ± 0.06 in developing xylem. The FPKM value of the fourth transcript was 4.33 ± 0.04 in phloem, 3.15 ± 0.07 in cambium, and 1.11 ± 0.05 in developing xylem (Table 4). Moreover, the first transcript included all exons, but the others lacked a part of the third exon in *NAC* (Figure S3). These results indicated that these genes produced different transcripts in different tissues.

**Table 4:** **Different transcripts' expression of candidate wood-related genes, MYB and NAC**.

	**MYB**		**NAC**			
	**Transcript 1**	**Transcript 2**	**Transcript 1**	**Transcript 2**	**Transcript 3**	**Transcript 4**
Phloem	142.74 ± 5.00	10.67 ± 0.39	220.87 ± 1.07	142.81 ± 5.14	0.81 ± 0.03	4.33 ± 0.04
Cambium	102.70 ± 3.29	13.48 ± 0.67	228.27 ± 4.11	111.61 ± 0.24	7.96 ± 0.27	3.15 ± 0.07
Developing xylem	36.92 ± 1.18	88.92 ± 1.18	102.90 ± 4.12	250.22 ± 8.25	2.15 ± 0.06	1.11 ± 0.05

## Discussion

Poplar trees are perennial plants with long lifespans and generation times; thus, they have to acclimate to changing environments. Along with having substantial variation in genotype and phenotype, poplar is widely recognized as an appropriate tree for epigenetic studies to examine the effects of epigenetic reprograming on differentiation, development, and in response to environmental stimuli (Vining et al., 2012). Taking advantage of poplar's high-quality genomic resources and extensive, highly elaborated tissue types, here we used the MSAP technique to interrogate the role DNA cytosine methylation plays in xylogenesis.

### Variation in DNA cytosine methylation pattern among different types of tissues and organs

In our study, genome-wide DNA cytosine methylation of the symmetrical tetranucleotide sequence 5′-CCGG site in different types of tissues and organs of poplar was determined using MSAP, which showed that 85% of the cytosines in 5′-CCGG are differentially methylated. The fully methylated sites (at an average level of 11.90%) were more frequently detected than the hemi-methylated sites (average 9.52%) among all tissues and organs studied, of which the total methylation level was 21.42%. DNA cytosine methylation varied greatly not only among different species but also among tissues. Also, the ratio of differential methylation in our study was much higher than observed in a previous study of Vining et al. (2012), which found that 33.7% of the *P. trichocarpa* genome was differentially methylated among seven tissues. The difference of *Populus* species, detecting methods of 5 mC, and the addition of three tissue or organ types might be the reason for the dramatic elevation of the differential methylation ratio. Our results revealed that variation of cytosine methylation among different tissues and organs is striking, and common methylated sites were very rare among the 10 tissue and organ types. We detected many fewer common methylation patterns (0.34%) in the 10 tissue and organ types compared with the results of Vining et al., who found that ~2% of the *P. trichocarpa* genome was methylated in all seven assayed tissues (Vining et al., 2012). Our study also detected specific cytosine methylation of tissues and organs at CpG-sites in *P. tomentosa*; in total, 26.81% of all the detected loci were specific cytosine methylation sites. Methylated cytosine could be detected in many regions of the genome, including intragenic and intergenic regions. We found that methylated cytosine tended to locate in the promoter and gene body, notably in exons. Previous studies reported that an estimated of 30% genes in *A. thaliana* (Zhang et al., 2006) and 16% of genes in rice (He et al., 2010) were extensively methylated. Furthermore, methylation within gene body regions occurred in nearly three-quarters of methylated genes examined by DNaseI-MethylDNA Immunoprecipitation followed by Illumina/Solexa sequencing (DNaseI-MeDIP-SEQ) in *P. trichocarpa* (Lafon-Placette et al., 2013). The finding in our study that the gene body was more frequently methylated in *P. tomentosa* could provide new evidence for gene body methylation, and this result also suggests that the phenomenon of gene-wide methylation was conserved in plants, especially within the same genus although in species belonging to different sections.

### The role of DNA methylation in xylogenesis

Although, various relative methylation levels existed in the 10 tissues, they did not significantly differ between xylem and non-xylem tissue types (*P* = 0.628 for relative hemi-methylation level, *P* = 0.254 for relative full methylation level, and 0.389 for relative non-methylation; Figure 2B). However, relative methylation did significantly differ (*P* < 0.05) between two tissue types, based on the relative methylation levels of 132 genes that have the same methylation pattern in developing xylem and mature xylem. This implies that the major cause of xylogenesis may be the methylation pattern of specific genes rather than the methylation level of the whole genome (Figure 2D).

The expression of genes was combined with the corresponding methylation pattern to test the effect of DNA methylation on wood formation by analyzing the relationship between gene expression and methylation. The results showed that methylation tends to suppress gene transcription, and hemi-methylation has higher power than full methylation (Figure 3). However, gene transcription did not significantly differ among phloem, cambium, developing xylem, and mature xylem (Figure 3) with similar methylation patterns in 61 (46.2%) of the candidate genes. Thus, phloem, cambium, and xylem (developing xylem and mature xylem) have physically distinct morphology in xylogenesis (Plomion et al., 2001), but they have similar expression profiles of candidate genes, and *MYB, NAC*, and *fasciclin-like AGP 13*, which are closely related to xylogenesis, have the same methylation pattern in the four vascular tissues. Among them, the NAC transcription factors NST1 and NST2, can regulate the biosynthesis of secondary walls (Mitsuda et al., 2005). A study on the Arabidopsis MYB transcription factor family revealed that MYB26 functions redundantly in regulating secondary wall thickening in the endothecium of anthers, and MYB26 can activate *NST1* and *NST2* (Yang et al., 2007). When cambium differentiates to phloem and developing xylem, the phloem cell has thick secondary wall with lots of fibers and little lignin while developing xylem cells have thin secondary walls (Zhong et al., 2007). These results indicate that MYB and NAC are major regulators of secondary wall biosynthesis, and also play an important role in xylogenesis. Here, bisulfite sequencing was used to examine phloem, cambium and developing xylem; those result revealed that although the same methylation pattern existed in the CCGG context, the methylation status on the whole gene differed in the three tissues. Analysis of bisulfite sequencing and RNA-seq revealed that the different location of cytosine methylation in the gene might affect alternative splicing, depending on the sequence context of the corresponding gene.

### The effect of alternative splicing

Protein structure prediction and functional annotation revealed that some genes produced different transcripts depending on their methylation state and the proteins encoded by these transcript may have distinct functions. The 3D-structure of the MYB-type HTH domain forms three α-helices. The second and third helices connect via a turn to make the helix-turn-helix motif. Helix 3 is termed the recognition helix as it binds the DNA major groove, like in other HTHs (Figure 5). The second transcript of *MYB* lacking the second exon encoding the part of the second and third helix loses the function of binding the DNA major groove in developing xylem, because the protein encoded by the second transcript has changed the structure and lost the binding domain. *NAC* also tends to express the second transcript without function in developing xylem, because the lost sequence encodes the linking part of the protein thus turning a whole functional protein into two useless halves (Figure S3). This suggests that the two major regulators in secondary wall biosynthesis act through expressing alternatively spliced transcripts to decrease the biosynthesis of secondary wall to inhibit cell elongation. In contrast, *MYB* and *NAC* mainly express the first transcript to promote secondary wall formation. Maunakea et al. (2013) found that alternative splicing may be affected by intragenic DNA methylation in exons. The methylation enhances exon recognition via recruitment of the multifunctional protein MeCP2, which can lead to exon skipping or maintain local histone hypoacetylation through the subsequent recruitment of histone deacetylase, whose inhibition also leads to exon skipping.

## Data archiving statement

Sequence data from this article have been deposited with the GenBank Data Library under the accession nos. KT972377–KT972586.

## Author contributions

DZ conceived and designed the research project. DC, TL, PL, YS, JC, MQ, and DZ helped with data analysis. QW conducted experiments and wrote the manuscript. All authors read and approved the manuscript.

### Conflict of interest statement

The authors declare that the research was conducted in the absence of any commercial or financial relationships that could be construed as a potential conflict of interest. The reviewer PZ and handling Editor declared their shared affiliation, and the handling Editor states that the process nevertheless met the standards of a fair and objective review.
