# Supplementary material for: The Role of DNA Methylation in Xylogenesis in Different Tissues of Poplar
Source: Front Plant Sci. 2016 Jul 12;7:1003. doi: 10.3389/fpls.2016.01003 (PMC4941658; doi:10.3389/fpls.2016.01003)
Supplement: Supplementary file 1 [file Table1.DOC]

**Table S1.** Statistical data of 135 selective primer combinations

| Primer  combinations | Count of  total bands | Count of  polymorphic markers | Range of polymorphic  fragments length (bp) |
| --- | --- | --- | --- |
| E32+H/M31 | 94 | 78 | 56-399 |
| E32+H/M33 | 89 | 66 | 55-462 |
| E32+H/M34 | 152 | 138 | 55-376 |
| E33+H/M33 | 78 | 61 | 55-423 |
| E33+H/M34 | 186 | 168 | 55-493 |
| E33+H/M44 | 72 | 60 | 55-325 |
| E33+H/M60 | 77 | 70 | 55-490 |
| E33+H/M63 | 57 | 57 | 57-294 |
| E33+H/M80 | 34 | 27 | 55-369 |
| E33+H/M82 | 36 | 36 | 56-407 |
| E33+H/M86 | 42 | 41 | 57-275 |
| E34+H/M33 | 73 | 69 | 57-444 |
| E34+H/M34 | 169 | 161 | 55-420 |
| E34+H/M44 | 51 | 51 | 55-273 |
| E34+H/M60 | 88 | 88 | 55-442 |
| E34+H/M63 | 31 | 31 | 55-315 |
| E34+H/M80 | 16 | 16 | 60-285 |
| E34+H/M82 | 35 | 35 | 62-451 |
| E34+H/M86 | 34 | 34 | 55-243 |
| E35+H/M33 | 14 | 14 | 59-160 |
| E35+H/M34 | 27 | 27 | 58-294 |
| E35+H/M44 | 60 | 60 | 55-325 |
| E35+H/M60 | 22 | 22 | 55-490 |
| E35+H/M63 | 75 | 75 | 55-321 |
| E35+H/M80 | 27 | 27 | 55-285 |
| E35+H/M82 | 19 | 19 | 58-268 |
| E35+H/M86 | 50 | 50 | 57-457 |
| E37+H/M31 | 129 | 123 | 55-467 |
| E37+H/M33 | 77 | 70 | 57-448 |
| E38+H/M31 | 53 | 45 | 57-453 |
| E38+H/M33 | 60 | 57 | 63-441 |
| E38+H/M34 | 15 | 15 | 57-256 |
| E38+H/M44 | 77 | 69 | 56-474 |
| E38+H/M46 | 27 | 27 | 57-440 |
| E38+H/M60 | 55 | 53 | 55-474 |
| E38+H/M65 | 16 | 16 | 56-470 |
| E38+H/M80 | 40 | 40 | 55-222 |
| E38+H/M82 | 54 | 43 | 56-282 |
| E38+H/M86 | 23 | 23 | 57-350 |
| E39+H/M31 | 118 | 97 | 55-477 |
| E39+H/M33 | 80 | 72 | 57-363 |
| E40+H/M31 | 34 | 34 | 57-453 |
| E40+H/M33 | 51 | 41 | 60-327 |
| E40+H/M34 | 12 | 12 | 57-283 |
| E40+H/M44 | 39 | 39 | 56-314 |
| E40+H/M46 | 11 | 11 | 55-239 |
| E40+H/M60 | 62 | 62 | 55-357 |
| E40+H/M65 | 18 | 18 | 56-213 |
| E40+H/M80 | 64 | 62 | 55-375 |
| E40+H/M82 | 41 | 34 | 56-215 |
| E40+H/M86 | 51 | 51 | 56-309 |
| E42+H/M31 | 106 | 95 | 57-453 |
| E42+H/M33 | 41 | 33 | 64-322 |
| E42+H/M34 | 79 | 74 | 55-312 |
| E42+H/M44 | 23 | 23 | 58-314 |
| E42+H/M46 | 54 | 48 | 55-435 |
| E42+H/M47 | 70 | 69 | 55-413 |
| E42+H/M60 | 52 | 52 | 55-357 |
| E42+H/M65 | 58 | 58 | 56-470 |
| E42+H/M80 | 22 | 22 | 55-151 |
| E42+H/M82 | 63 | 48 | 56-313 |
| E42+H/M86 | 63 | 63 | 61-325 |
| E44+H/M31 | 106 | 100 | 55-488 |
| E44+H/M33 | 51 | 38 | 55-374 |
| E45+H/M31 | 129 | 106 | 55-476 |
| E45+H/M33 | 75 | 55 | 57-482 |
| E46+H/M31 | 159 | 147 | 55-453 |
| E46+H/M33 | 63 | 49 | 57-447 |
| E47+H/M31 | 114 | 105 | 56-367 |
| E47+H/M33 | 73 | 60 | 57-404 |
| E49+H/M31 | 111 | 91 | 55-386 |
| E49+H/M33 | 60 | 53 | 57-444 |
| E50+H/M31 | 107 | 90 | 55-360 |
| E50+H/M33 | 93 | 86 | 55-317 |
| E51+H/M31 | 102 | 96 | 57-310 |
| E51+H/M33 | 82 | 71 | 57-220 |
| E53+H/M31 | 100 | 95 | 55-453 |
| E53+H/M33 | 32 | 29 | 57-200 |
| E54+H/M31 | 106 | 88 | 55-412 |
| E54+H/M33 | 172 | 157 | 57-360 |
| E55+H/M31 | 99 | 89 | 55-453 |
| E55+H/M33 | 169 | 164 | 55-396 |
| E57+H/M31 | 131 | 113 | 55-467 |
| E57+H/M33 | 178 | 169 | 55-362 |
| E58+H/M31 | 131 | 116 | 55-453 |
| E58+H/M33 | 200 | 91 | 56-500 |
| E58+H/M44 | 172 | 96 | 55-499 |
| E58+H/M47 | 75 | 44 | 66-498 |
| E58+H/M60 | 96 | 70 | 55-499 |
| E58+H/M80 | 185 | 109 | 55-500 |
| E58+H/M86 | 155 | 119 | 59-500 |
| E59+H/M31 | 129 | 108 | 55-482 |
| E59+H/M33 | 141 | 113 | 56-455 |
| E60+H/M31 | 112 | 102 | 55-418 |
| E60+H/M33 | 151 | 138 | 55-435 |
| E63+H/M31 | 131 | 111 | 55-464 |
| E63+H/M33 | 162 | 142 | 55-435 |
| E65+H/M31 | 116 | 98 | 55-496 |
| E65+H/M33 | 198 | 185 | 55-386 |
| E66+H/M31 | 104 | 96 | 55-453 |
| E66+H/M33 | 159 | 158 | 55-400 |
| E67+H/M31 | 118 | 108 | 57-453 |
| E67+H/M33 | 154 | 141 | 57-498 |
| E68+H/M31 | 101 | 86 | 55-454 |
| E68+H/M33 | 173 | 160 | 55-408 |
| E76+H/M31 | 90 | 77 | 55-473 |
| E76+H/M33 | 173 | 162 | 55-426 |
| E80+H/M31 | 85 | 74 | 57-440 |
| E80+H/M33 | 148 | 146 | 55-314 |
| E81+H/M31 | 82 | 65 | 55-400 |
| E81+H/M33 | 153 | 135 | 55-480 |
| E83+H/M31 | 63 | 37 | 57-356 |
| E83+H/M33 | 99 | 86 | 55-257 |
| E86+H/M31 | 79 | 55 | 57-379 |
| E86+H/M33 | 168 | 167 | 57-388 |
| E87+H/M31 | 85 | 69 | 55-472 |
| E87+H/M33 | 170 | 154 | 56-417 |
| E88+H/M31 | 57 | 48 | 55-355 |
| E88+H/M33 | 167 | 146 | 55-418 |
| E90+H/M31 | 54 | 40 | 57-453 |
| E90+H/M33 | 200 | 88 | 56-500 |
| E90+H/M44 | 172 | 83 | 55-499 |
| E90+H/M47 | 76 | 39 | 66-498 |
| E90+H/M60 | 96 | 38 | 55-499 |
| E90+H/M80 | 185 | 99 | 55-500 |
| E90+H/M86 | 155 | 86 | 59-500 |
| E91+H/M31 | 59 | 50 | 57-453 |
| E91+H/M33 | 200 | 80 | 56-500 |
| E91+H/M44 | 172 | 73 | 55-499 |
| E91+H/M47 | 75 | 48 | 66-498 |
| E91+H/M60 | 96 | 39 | 55-499 |
| E91+H/M80 | 185 | 97 | 55-500 |
| E91+H/M86 | 155 | 102 | 59-500 |
| E92+H/M31 | 51 | 28 | 55-368 |
| E92+H/M33 | 204 | 183 | 55-458 |
| Total | 12581 | 10316 |  |
