# Supplementary material for: The Role of DNA Methylation in Xylogenesis in Different Tissues of Poplar
Source: Front Plant Sci. 2016 Jul 12;7:1003. doi: 10.3389/fpls.2016.01003 (PMC4941658; doi:10.3389/fpls.2016.01003)
Supplement: Supplementary file 2 [file Table2.DOC]

**Table S2.** Sequences of adapters and primers

| Adapters/Primers | | | Sequence |
| --- | --- | --- | --- |
| EcoRⅠadapters | | AdapterⅠ | 5'-CTCGTAGACTGCGTACC-3' |
|  |  | AdapterⅡ | 5'-AATTGGTACGCAGTC-3' |
| *Hpa*Ⅱ/*Msp*Ⅰ adapters | | AdapterⅢ | 5'-GATCATGAGTCCTGCT-3' |
|  |  | AdapterⅣ | 5'-CGAGCAGGACTCATGA-3' |
| Pre-amplification primers | | *Eco*RⅠ00 | 5'-GACTGCGTACCAATTC-3' |
|  |  | HpaⅡ/MspⅠ00 | 5'-ATCATGAGTCCTGCTCGG-3' |
| Selective  amplification  primers | *Eco*RⅠprimers | *Eco*RI32 | 5'-GACTGCGTACCAATTCAAC-3' |
|  |  | *Eco*RI33 | 5'-GACTGCGTACCAATTCAAG-3' |
|  |  | *Eco*RI34 | 5'-GACTGCGTACCAATTCAAT -3' |
|  |  | *Eco*RI35 | 5'-GACTGCGTACCAATTCACA -3' |
|  |  | *Eco*RI37 | 5'-GACTGCGTACCAATTCACG -3' |
|  |  | *Eco*RI38 | 5'-GACTGCGTACCAATTCACT -3' |
|  |  | *Eco*RI39 | 5'-GACTGCGTACCAATTCAGA -3' |
|  |  | *Eco*RI40 | 5'-GACTGCGTACCAATTCAGC -3' |
|  |  | *Eco*RI42 | 5'-GACTGCGTACCAATTCAGT -3' |
|  |  | *Eco*RI44 | 5'-GACTGCGTACCAATTCATC -3' |
|  |  | *Eco*RI45 | 5'-GACTGCGTACCAATTCATG -3' |
|  |  | *Eco*RI46 | 5'-GACTGCGTACCAATTCATT -3' |
|  |  | *Eco*RI47 | 5'-GACTGCGTACCAATTCCAA -3' |
|  |  | *Eco*RI49 | 5'-GACTGCGTACCAATTCCAG -3' |
|  |  | *Eco*RI50 | 5'-GACTGCGTACCAATTCCAT -3' |
|  |  | *Eco*RI51 | 5'-GACTGCGTACCAATTCCCA -3' |
|  |  | *Eco*RI53 | 5'-GACTGCGTACCAATTCCCG -3' |
|  |  | *Eco*RI54 | 5'-GACTGCGTACCAATTCCCT -3' |
|  |  | *Eco*RI55 | 5'-GACTGCGTACCAATTCCGA -3' |
|  |  | *Eco*RI57 | 5'-GACTGCGTACCAATTCCGG -3' |
|  |  | *Eco*RI58 | 5'-GACTGCGTACCAATTCCGT -3' |
|  |  | *Eco*RI59 | 5'-GACTGCGTACCAATTCCTA -3' |
|  |  | *Eco*RI60 | 5'-GACTGCGTACCAATTCCTC -3' |
|  |  | *Eco*RI63 | 5'-GACTGCGTACCAATTCGAA -3' |
|  |  | *Eco*RI65 | 5'-GACTGCGTACCAATTCGAG -3' |
|  |  | *Eco*RI66 | 5'-GACTGCGTACCAATTCGAT -3' |
|  |  | *Eco*RI67 | 5'-GACTGCGTACCAATTCGCA -3' |
|  |  | *Eco*RI68 | 5'-GACTGCGTACCAATTCGCC -3' |
|  |  | *Eco*RI76 | 5'-GACTGCGTACCAATTCGTC -3' |
|  |  | *Eco*RI80 | 5'-GACTGCGTACCAATTCTAC -3' |
|  |  | *Eco*RI81 | 5'-GACTGCGTACCAATTCTAG -3' |
|  |  | *Eco*RI83 | 5'-GACTGCGTACCAATTCTCA -3' |
|  |  | *Eco*RI86 | 5'-GACTGCGTACCAATTCTCT -3' |
|  |  | *Eco*RI87 | 5'-GACTGCGTACCAATTCTGA -3' |
|  |  | *Eco*RI88 | 5'-GACTGCGTACCAATTCTGC -3' |
|  |  | *Eco*RI90 | 5'-GACTGCGTACCAATTCTGT -3' |
|  |  | *Eco*RI91 | 5'-GACTGCGTACCAATTCTTA -3' |
|  |  | *Eco*RI92 | 5'-GACTGCGTACCAATTCTTC -3' |
|  | H/M primer  （5'Biotin） | H/M31 | 5'-ATCATGAGTCCTGCTCGGAAA-3' |
|  |  | H/M33 | 5'-ATCATGAGTCCTGCTCGGAAG -3' |
|  |  | H/M34 | 5'-ATCATGAGTCCTGCTCGGAAT -3' |
|  |  | H/M44 | 5'- ATCATGAGTCCTGCTCGGATC -3' |
|  |  | H/M46 | 5'- ATCATGAGTCCTGCTCGGATT -3' |
|  |  | H/M47 | 5'- ATCATGAGTCCTGCTCGGCAA -3' |
|  |  | H/M60 | 5'- ATCATGAGTCCTGCTCGGCTC -3' |
|  |  | H/M63 | 5'- ATCATGAGTCCTGCTCGGGAA -3' |
|  |  | H/M65 | 5'- ATCATGAGTCCTGCTCGGGAG -3' |
|  |  | H/M80 | 5'- ATCATGAGTCCTGCTCGGTAC-3' |
|  |  | H/M82 | 5'- ATCATGAGTCCTGCTCGGTAT -3' |
|  |  | H/M86 | 5'- ATCATGAGTCCTGCTCGGTCT -3' |
