# Supplementary material for: The Role of DNA Methylation in Xylogenesis in Different Tissues of Poplar
Source: Front Plant Sci. 2016 Jul 12;7:1003. doi: 10.3389/fpls.2016.01003 (PMC4941658; doi:10.3389/fpls.2016.01003)
Supplement: Supplementary file 3 [file Table3.DOCX]

**Table S3.** Information on bisulfite sequencing of candidate genes

| **Alias** | **Gene model** | **Gene site** | **Fragment site**^a^ | **Sequence** | **Efficiency (%)** |
| --- | --- | --- | --- | --- | --- |
| MYB | Potri.016G047900 | **Chr16:3044137-3047342** | **Chr16:**3044015-3044932 | F: TACTTTGCGTGGCTTGTA  R: AGACCTTTATCGGACTTG | 99.7~108.1 |
|  |  |  | **Chr16:**3044811-3045966 | F: CTTTGACAAGTCCGATAA  R: CAAGTGTTTGAAGGGTAG | 98.5~101.7 |
|  |  |  | **Chr16:**3045811-3046269 | F: AGAAGTCCAACTTGCCTGTA  R: CCCTGATTTGAAACCCAC | 97.3~101.9 |
|  |  |  | **Chr16:**3046235-3047415 | F: CGGACGAGTCAACATTTA  R: GAGTGCAGCATACAGAGC | 99.7~108.1 |
| NAC | Potri.001G404100 | **Chr01:42547278-42549008** | **Chr01:**42547153-42548123 | F: AACGGTGACAAGCATAAA  R: AATACCCATTCGTCCAAC | 97.9~106.4 |
|  |  |  | **Chr01:**42548056-42548445 | F: TTTGATACGCCTCAATAT  R: ATGGCATCTAATCCTCTA | 98.8~105.6 |
|  |  |  | **Chr01:**42548411-42549325 | F: AACTCATTTGACCCGTAT  R: TTCGAACACTCACGTAAA | 97.9~102.1 |

^a^ The information of fragment site presents the length of fragment amplified by primer combination.
