# Supplementary material for: The Role of DNA Methylation in Xylogenesis in Different Tissues of Poplar
Source: Front Plant Sci. 2016 Jul 12;7:1003. doi: 10.3389/fpls.2016.01003 (PMC4941658; doi:10.3389/fpls.2016.01003)
Supplement: Supplementary file 4 [file Table4.DOCX]

**Table S4.** Information of realtime-PCR primer sequences for 132 candidate genes

| **Fragment** | **Gene model** | **Sequence (5'--3')** | **Efficiency (%)** |
| --- | --- | --- | --- |
| MSAP fragment1 | Potri.014G155200 | F: GTTGGAAGGTCTGGGATA  R: GGTAACAGGAAAGCGAAA | 98.9~105.4 |
| MSAP fragment2 | Potri.014G008900 | F: TCAGGCAGGGCTGGTTTA  R: CTCGCTACAGTCCTCATCATTT | 98.2~105.2 |
| MSAP fragment24 | Potri.001G424200 | F: CATTTCCCATCCAAGATA  R: GAGTGCAGCATACAGAGC | 95.9~104.9 |
| MSAP fragment42 | Potri.016G047900 | F: GGCTTACCTCATCCTGTG  R: CATTCCCTTCTCCTTTCA | 98.8~105.6 |
| MSAP fragment58 | Potri.009G041200 | F: GCATCACTCATATATCA  R: CTACCATTTCTCCGTCCT | 98.9~105.4 |
| MSAP fragment59 | Potri.009G054300 | F: TTTCGCTGTCAAAGTAGAAC  R: ATTGCCATGAGTTCCT | 97.9~106.4 |
| MSAP fragment60 | Potri.009G067300 | F: ACTACTGTGCCCTTTGAC  R: TTCGTTGACTTGAATATCTG | 98.8~105.6 |
| MSAP fragment61 | Potri.009G123100 | F: GCTGTTGACCCTCTGGTG  R: CCCTTGCTGCTTATCCCT | 99.7~108.1 |
| MSAP fragment62 | Potri.009G153200 | F: ATCTGAGAATTAAGTCGTTTGA  R: TGGGCTAAGATACCGTCA | 98.5~101.7 |
| MSAP fragment63 | Potri.001G399500 | F: CTCAGGTTCTACCCTCCC  R: GATCTTGTGCCCTCTTCC | 98.5~101.7 |
| MSAP fragment64 | Potri.001G170300 | F: TAACTTCCTTTCCTCCTA  R: CTCTAAACATACTCCCATC | 97.3~101.9 |
| MSAP fragment65 | Potri.001G168200 | F: AGCGGCACTGTTGGATAG  R: GGAATAGAAGGCGAATGG | 97.5~100.7 |
| MSAP fragment66 | Potri.001G275500 | F: CTACTACAGTCGGGCACA  R: CTTCACAACAGAGGGATC | 97.9~102.1 |
| MSAP fragment67 | Potri.001G344600 | F: ACGGACCAGTTTCCATCA  R: TCCCAGGTATCCTAAGTTCA | 97.9~106.4 |
| MSAP fragment69 | Potri.001G404100 | F: CTAACTCATTTGACCCGTAT  R: CTCTAGCAACCCAGAAGG | 98.2~105.2 |
| MSAP fragment70 | Potri.001G418600 | F: TTGCCTATGTTTGGTTGT  R: TTGTGCCAGTTTGGTATT | 95.9~104.9 |
| MSAP fragment71 | Potri.001G129100 | F: ATTGAGTCGCCGAGTGGA  R: CTTGAGAACCCGCCAGAA | 98.8~105.6 |
| MSAP fragment72 | Potri.001G040500 | F: GCTGCTTGTTGGTATCTG  R: ATCCTTAGTCGCATTTCC | 98.9~105.4 |
| MSAP fragment73 | Potri.001G007300 | F: AGTTTAGCAGCGGTGAAG  R: AGTACAAATCCTGGGAGG | 97.2~103.1 |
| MSAP fragment93 | Potri.001G435900 | F: TGGCTCCTGAGTTGTTGT  R: CTTGAGTGGCTCTTCCCT | 99.1~101.9 |
| MSAP fragment110 | Potri.005G140200 | F: ATCTCCACCATAACCTAA  R: ATAAACCAGCCTACAAAC | 99.7~108.1 |
| MSAP fragment111 | Potri.005G140300 | F: CTTGTCGCACAGGGTTCT  R: ATCCGATTCGTATCCATAAA | 98.5~101.7 |
| MSAP fragment117 | Potri.003G077900 | F: AACTGACCACTCCCTGAC  R: GTGCGTTTCTTACATTACATAG | 97.3~101.9 |
| MSAP fragment120 | Potri.005G148400 | F: TTTAGGAGGTTAGTGGAGG  R: GGGCTCGTACTTAACAGCAT | 99.7~108.1 |
| MSAP fragment130 | Potri.016G119300 | F: GGGGTATAGTATGAGTTATG  R: TTAGTTACTCGGAGTTGTG | 97.9~106.4 |
| MSAP fragment131 | Potri.016G119400 | F: GTGCTTGGTTTGCTGGTG  R: TGGTTCTGCGACATCCTG | 98.8~105.6 |
| MSAP fragment140 | Potri.015G132800 | F: GTCACCGAGTCAATCCAA  R: TCCCTCCTCTGTCTGTAATC | 97.9~102.1 |
| MSAP fragment141 | Potri.008G026100 | F: GTTTGAGCGACATAGTTG  R: TGAAGGGAGACTCTGGTA | 99.7~108.1 |
| MSAP fragment146 | Potri.003G023300 | F: TGTCATTGTTTGGTGGAA  R: TAACTTTAGGGCAGAGGC | 98.5~101.7 |
| MSAP fragment147 | Potri.006G129200 | F: TTGAACTGAGGTGGATTA  R: ATCTACAGGATCGGAATT | 97.3~101.9 |
| MSAP fragment148 | Potri.005G228400 | F: AAAACAGAGGAGCTTCAA  R: CACTGGGTTCTTCATCGT | 97.5~100.7 |
| MSAP fragment150 | Potri.004G177600 | F: CAAGAACATCAAAAGCAG  R: TTATTAAAAGAAGGTGGC | 98.2~105.2 |
| MSAP fragment152 | Potri.001G405400 | F: CGAGTAATTGATATTGCTCCTG  R: TTCGCAGACCTACCAAACC | 95.9~104.9 |
| MSAP fragment154 | Potri.007G097100 | F: ACTAGGAAACAAGGAGGGA  R: CCTTTCACCTAAGCCCAC | 98.8~105.6 |
| MSAP fragment166 | Potri.009G058200 | F: CGGTAGGACGCATCTTCT  R: GCTCGTGTCAATCCCTGT | 98.9~105.4 |
| MSAP fragment169 | Potri.003G124100 | F: CTGCTGGTTAGTGTTGCT  R: CATTGCCATTCTCAGTCC | 97.9~102.1 |
| MSAP fragment170 | Potri.010G184500 | F: GAGTTTAGCCATCGTTTC  R: AAGATCCTGTCCGTATTG | 99.7~108.1 |
| MSAP fragment179 | Potri.013G100200 | F: CCTGATACATTTCCCTTTG  R: CCAGCTTGGGTACTTGAC | 98.5~101.7 |
| MSAP fragment186 | Potri.009G032000 | F: AAACACCAGCAGGCAGAG  R: ACGAGACAGCACCATTCC | 97.3~101.9 |
| MSAP fragment187 | Potri.009G032100 | F: TGTAGCGATTGATGATTTG  R: AGAACTGCTCCAGGGTGA | 97.5~100.7 |
| MSAP fragment188 | Potri.006G136400 | F: AACCTGCTCTGAGATGCT  R: TCCTTTCCTCCAAACACT | 98.2~105.2 |
| MSAP fragment212 | Potri.017G113800 | F: GCAAGAAAATACTTCAAAC  R: TTCCCAATACCAATAACA | 95.9~104.9 |
| MSAP fragment216 | Potri.001G211300 | F: AGAACGAAAGGAGGTTTA  R: GATGATTACTCGCTACATTA | 98.8~105.6 |
| MSAP fragment222 | Potri.001G103300 | F: CGATGAAGGGAACAGTAG  R: GCTCTATGTCCACGAAGT | 98.9~105.4 |
| MSAP fragment225 | Potri.004G024500 | F: CGATAGGGAGTAGGTGTA  R: TGGGCATTCATTCAAAAT | 99.7~108.1 |
| MSAP fragment228 | Potri.005G040600 | F: TTTAGTGGCTGATCCCTT  R: AGACTTGGTGCATGTTTA | 98.5~101.7 |
| MSAP fragment231 | Potri.014G015100 | F: CCTGAAGTGGTGGGAGAA  R: AATCAAAGGGCAGGGAAC | 97.3~101.9 |
| MSAP fragment233 | Potri.002G156200 | F: GATTTAGCCTAGCATACAC  R: CAGTTTCCCATCAGCATT | 99.7~108.1 |
| MSAP fragment234 | Potri.016G006500 | F: GCTTTCATGGGTGTTCTA  R: TTCATTGCCAAGTTGTTG | 97.9~106.4 |
| MSAP fragment235 | Potri.010G018800 | F: CAGAGGCTTCTGGGAACT  R: CCAGTGAAAGCAGGATGA | 99.7~108.1 |
| MSAP fragment172 | Potri.006G255100 | F: GATTCACCCAGGAAATGA  R: GTACCCTAACCTTCACGT | 98.8~105.6 |
| MSAP fragment208 | Potri.003G078000 | F: TACTGTGAATTGCGTGTT  R: GTGACCTTCTGAGTTTGG | 98.2~105.2 |
| MSAP fragment4 | Potri.002G048100 | F: TTTTCTTCCCTGGATGGT  R: CTCGGTTGGTATTCTTTGCT | 95.9~104.9 |
| MSAP fragment6 | Potri.003G110200 | F: ATTGAGGAGTAGGTGCTG  R: CATCTTCTTGTGCGGTAA | 98.1~103.9 |
| MSAP fragment9 | Potri.001G330300 | F: GATGTTAGGCTTTGTGGT  R: ATGTAAGGTGGTTCAGTT | 97.9~102.1 |
| MSAP fragment31 | Potri.006G137600 | F: TTTGGCTGGCACGACTTA  R: AGGGCTGAATGCTTCTGATA | 99.7~108.1 |
| MSAP fragment32 | Potri.008G179600 | F: AGAATGATGAATGGGATG  R: GATAATGCGAGAAAGGAA | 98.5~101.7 |
| MSAP fragment53 | Potri.016G068900 | F: GGGAAAGGGAGTTGTATC  R: AAGCAGCAGTAGAGGTTG | 97.3~101.9 |
| MSAP fragment57 | Potri.009G026700 | F: CATACATAGATGCGACAA  R: CACCAGCTTATTCAACTGAC | 97.5~100.7 |
| MSAP fragment92 | Potri.001G343500 | F: ACAGCGAAAAGGAGGAAG  R: CTGCACCACAGGCAATAA | 97.9~102.1 |
| MSAP fragment94 | Potri.001G458900 | F: TATCTAAACCCACGAATG  R: TCTGATTGAAGAAGTCCC | 97.9~106.4 |
| MSAP fragment108 | Potri.005G160900 | F: AGGAGCCGAAGTGGAGGT  R: CTTTGATGGAACCGAGCC | 98.2~105.2 |
| MSAP fragment109 | Potri.001G431800 | F: GTTCCAGCTTTGACTATGT  R: AAGAAACCACTACCCATT | 95.9~104.9 |
| MSAP fragment118 | Potri.005G255400 | F: CAAGCCAAAGACCACAAC  R: TTCTAAGCCTTCGCAATC | 98.8~105.6 |
| MSAP fragment119 | Potri.005G148300 | F: AAGACTACTATGGTGGGA  R: TCGTGCTTTACAGATCAA | 98.9~105.4 |
| MSAP fragment134 | Potri.019G083000 | F: TAGTGTTTGTGCCCTGAT  R: CAGTTAGAGCCATTGAGG | 97.2~103.1 |
| MSAP fragment143 | Potri.002G105000 | F: AGGAAGTTTGCTTTTGAA  R: CAGTCGCATCCTACATTA | 99.1~101.9 |
| MSAP fragment209 | Potri.004G155000 | F: GGGGAAACCAGATTGATA  R: CAGCATAGAACAGGCACA | 99.7~108.1 |
| MSAP fragment230 | Potri.017G114800 | F: TTCTTGGCTTGATGAACG  R: AATGGCAGCCCTTTGTTA | 98.5~101.7 |
| MSAP fragment232 | Potri.017G063900 | F: TTTTCGTACAGATGCTTTG  R: CTCTTGACACGGAACCTA | 97.3~101.9 |
| MSAP fragment207 | Potri.003G121800 | F: GGTGACAGTGCTGGTTTC  R: CCATATTTCATTTCTAGGGT | 99.7~108.1 |
| MSAP fragment164 | Potri.003G192400 | F: AATGTAACGCCGATAAAC  R: TAATCCGAGTACGCTTCT | 97.9~106.4 |
| MSAP fragment82 | Potri.001G188300 | F: GGCATTTAGGTTAGAAGT  R: CAAATATCATAAGGCAGA | 98.8~105.6 |
| MSAP fragment107 | Potri.005G136000 | F: TAGAAGTTTGAAAGGAGGAG  R: GTTTGCAGGAAAGGAATA | 97.9~102.1 |
| MSAP fragment121 | Potri.003G095400 | F: AGTGAGCAGCAGCCTTCC  R: CGGCGAGTAATCAAGTGGT | 99.7~108.1 |
| MSAP fragment167 | Potri.009G058400 | F: TAAGCCTAACACTGTCACTT  R: CATTCCCAGAAACAACTC | 98.5~101.7 |
| MSAP fragment171 | Potri.006G255000 | F: ATGCTGGGAAATTGTGAT  R: TACAGAACCGTCCATCCA | 97.3~101.9 |
| MSAP fragment182 | Potri.011G049700 | F: CAAGACTGCGATTACAAA  R: GAAATACTGGGAACCATC | 97.5~100.7 |
| MSAP fragment203 | Potri.012G076500 | F: ATCAATTTGTTTGCCTCC  R: TTCCTCACTTCTCCGTTT | 98.2~105.2 |
| MSAP fragment223 | Potri.001G180200 | F: CCTTTTGTTATGGCTTAC  R: TATCTTCATTGCTTTGGA | 95.9~104.9 |
| MSAP fragment43 | Potri.010G028100 | F: CAGCCATAAATCCACTACCA  R: CCAGTCCTCCTCCCATCA | 98.8~105.6 |
| MSAP fragment78 | Potri.001G157900 | F: TCATAAGACTATTGCTCCAA  R: TAAACTCCCAAAGAACCT | 98.9~105.4 |
| MSAP fragment79 | Potri.001G161700 | F: ACACTGCTGTTTTGTTGA  R: TGCGAATTTAGATACTGG | 97.9~102.1 |
| MSAP fragment84 | Potri.001G208600 | F: CACTGCTGTTTTGTTGAC  R: TGCGAATTTAGATACTGG | 99.7~108.1 |
| MSAP fragment102 | Potri.010G098400 | F: CTTGATATTCCCTCCCTGTG  R: GCAACCCTTTCCCTTACC | 98.5~101.7 |
| MSAP fragment136 | Potri.019G005000 | F: GCGTACAAGGCAGAGGAT  R: TTCAGCCCAGTTACAGCA | 97.3~101.9 |
| MSAP fragment156 | Potri.010G235800 | F: TATCTGGTGGAGGTGGTA  R: CGTAGGCAAAGGTAAACA | 97.5~100.7 |
| MSAP fragment157 | Potri.010G235900 | F: TTACTACGGGAAGGACTG  R: GACCAATATGCCAAAGAT | 98.2~105.2 |
| MSAP fragment158 | Potri.012G068800 | F: AGAGATGATGAACCATGCCGTTAAG  R: CTTAACGGCATGGTTCATCATCTCT | 95.9~104.9 |
| MSAP fragment159 | Potri.014G009000 | F: GCAGGAAGGGTTTCAAGT  R: TTGGTTTTGTGGAAGGAA | 98.8~105.6 |
| MSAP fragment160 | Potri.016G066600 | F: GTGAATAGGCTGGCTGTA  R: AAGCATCGCATAAAAGAA | 98.9~105.4 |
| MSAP fragment168 | Potri.007G033400 | F: GGGGTTAGTTGTTGAAGT  R: GGACAGCCAAAGGTATTG | 98.2~105.2 |
| MSAP fragment174 | Potri.001G024600 | F: GCACAGCTTGAGCTATTG  R: GGAAATCCATCCTGGTTA | 95.9~104.9 |
| MSAP fragment176 | Potri.014G100000 | F: GATTATCATGTGGGAGTTGAGGTCT  R: AGACCTCAACTCCCACATGATAATC | 98.8~105.6 |
| MSAP fragment180 | Potri.006G124800 | F: TTTCCTTTATCCGTTCAATC  R: CTACAACAGCCACGCACT | 98.9~105.4 |
| MSAP fragment200 | Potri.006G074700 | F: GTTATCAAGGGTTACCGA  R: ATGATGATGACGACCAAA | 97.9~106.4 |
| MSAP fragment224 | Potri.001G401300 | F: AGAAACTGCCCTGATGAT  R: CTCGTAATCAGAAGGCTC | 98.8~105.6 |
| MSAP fragment236 | Potri.010G029100 | F: CCTTGTAAATAAAGACCCTG  R: CCTTCAATTCCTTACCC | 99.7~108.1 |
| MSAP fragment237 | Potri.010G034300 | F: TGCTTCTTCATACCACAT  R: GTACCCAGAGTAACACCC | 98.5~101.7 |
| MSAP fragment238 | Potri.010G106200 | F: TCAAGAACTCCGTGAACT  R: GACTGCCACAATCCTTTC | 97.3~101.9 |
| MSAP fragment241 | Potri.010G119300 | F: AGCACCCGTCCGAAAGAG  R: CCGTTCAACGAAACAAGG | 97.9~102.1 |
| MSAP fragment122 | Potri.007G052600 | F: TAATCAAAGGGGAGAAAT  R: AGAATAAGCATCCACTGT | 99.7~108.1 |
| MSAP fragment162 | Potri.005G036400 | F: TGAAAGGTTGAGGTAGTT  R: TTTTAATGGTAGGGGTAA | 98.5~101.7 |
| MSAP fragment239 | Potri.010G114400 | F: GGCACTTTAGGCACTGAT  R: TCTGGGTAACTTGTGACTCTTT | 97.3~101.9 |
| MSAP fragment116 | Potri.001G314100 | F: GCAGGAGAATGCAGAAGA  R: AACCGAACAACTGAAGAAAG | 97.5~100.7 |
| MSAP fragment149 | Potri.002G034700 | F: GCAGTAAACCAGTGCGTCTG  R: AGCCCTCTTAGGCATCGT | 98.2~105.2 |
| MSAP fragment151 | Potri.004G177700 | F: AAAACCCTGAACAGATTG  R: AAGCCTCGAAGAAGATTA | 95.9~104.9 |
| MSAP fragment173 | Potri.003G201000 | F: GTTTAACTCACCCAACCG  R: CGAAATACATCAAGGGAATA | 97.9~106.4 |
| MSAP fragment184 | Potri.004G087600 | F: ACTGCATTATCTCATCTC  R: CCTTATAGTTTATCATCG | 98.2~105.2 |
| MSAP fragment191 | Potri.013G155100 | F: GCAGAATGGAGCTATCAA  R: ATCCCCTTTTCAGGTTTA | 95.9~104.9 |
| MSAP fragment201 | Potri.006G158700 | F: CCTATGTGGGCTATCGTG  R: TTCCGCATTCTTATCCTC | 98.8~105.6 |
| MSAP fragment202 | Potri.011G082500 | F: CTTAGCCGTGTTAGGTTT  R: GTTTGGCAATATGGTTTC | 98.9~105.4 |
| MSAP fragment220 | Potri.003G012400 | F: TTGACTTCTCCTCCTTAA  R: AGTCTTCGTCGTGTTTAG | 99.7~108.1 |
| MSAP fragment229 | Potri.005G079400 | F: CATGGGCTGTGGTGAAAC  R: ATTCTCCACGGCAGGATT | 98.5~101.7 |
| MSAP fragment75 | Potri.001G096400 | F: TTTGCAGATTGGGTAAGA  R: TGATGCCATGTGATGAAC | 97.3~101.9 |
| MSAP fragment205 | Potri.014G037900 | F: CCCAACAGAGTTCAAGCC  R: TAGCCCAGCAGTTCCAGT | 99.7~108.1 |
| MSAP fragment206 | Potri.007G117800 | F: CCTTCAGGACATACCAAAGC  R: CGCAGCCACAGGTCATAC | 98.2~105.2 |
| MSAP fragment46 | Potri.016G071700 | F: AGCCCTCCTTTATTTATTTG  R: TTATGTCCAGCCTCCTCT | 95.9~104.9 |
| MSAP fragment190 | Potri.013G154900 | F: GAAGACAGTGAGCGTGAC  R: CTACCTCCATATCCTACAA | 97.9~106.4 |
| MSAP fragment11 | Potri.001G062100 | F: ATGGCATTTGGTTATTAT  R: TTTGAAGGTTGAAAGAGT | 98.8~105.6 |
| MSAP fragment12 | Potri.005G172200 | F: AGGCATTATTTAGGATTGG  R: AGAGCACGGCAGCAGTAG | 98.2~105.2 |
| MSAP fragment23 | Potri.002G171600 | F: GTTGACGGACAAGCAGTT  R: AACAATGTTTGTGGGAAG | 95.9~104.9 |
| MSAP fragment36 | Potri.009G133400 | F: ACTGGTTTACAGGGGTGG  R: ATGAAAGAAAAGGATGGT | 97.9~102.1 |
| MSAP fragment125 | Potri.001G439400 | F: TTTCGTGGGTATAAATGT  R: TTAAAGGAGACAAAATCAGT | 99.7~108.1 |
| MSAP fragment144 | Potri.002G105100 | F: ATTCCTATGTTGCCCGTAT  R: CAGGTGGGTGTTTGTCCT | 98.5~101.7 |
| MSAP fragment145 | Potri.003G023000 | F: GATCCGAAGATTATTCACC  R: AAAGCCCTTTGTCCAGTA | 97.3~101.9 |
| MSAP fragment175 | Potri.002G172400 | F: TTGCTCGGCTGCATCTGT  R: TAGGACGCCCAAACGCTA | 97.5~100.7 |
| MSAP fragment138 | Potri.001G162200 | F: GCTAGAAGGGTCAGTGGT  R: CAACAGAACGTGGTATGG | 99.7~108.1 |
| MSAP fragment153 | Potri.005G058300 | F: CGTATAGCGATTGAGAAA  R: AACGGTGAATACATAGACAG | 98.5~101.7 |
| MSAP fragment16 | Potri.015G003100 | F: CAAATTACCTCCATAGC  R: CTGGCATAAGATCAAGAA | 97.3~101.9 |
| MSAP fragment28 | Potri.010G078600 | F: CCACTTTCCCTGTCTTCG  R: ATCACCTTCCATGCCAAC | 98.2~105.2 |
| MSAP fragment219 | Potri.003G027400 | F: CTTGATTAAAGCGGTTAG  R: TTCCCATGTTGTGAGAAA | 95.9~104.9 |
