# Supplementary material for: The Role of DNA Methylation in Xylogenesis in Different Tissues of Poplar
Source: Front Plant Sci. 2016 Jul 12;7:1003. doi: 10.3389/fpls.2016.01003 (PMC4941658; doi:10.3389/fpls.2016.01003)
Supplement: Supplementary file 5 [file Table5.DOC]

**Table S5.** Characterization of the polymorphic MSAP fragments

| Fragment | Length (bp) | Reference gene ID | Methylation location | Protein homolog |
| --- | --- | --- | --- | --- |
| MF4 | 64 | Potri.002G048100 | exon |  |
| MF6 | 178 | Potri.003G110200 | 3' flanking region |  |
| MF7 | 178 | Potri.009G081500 | promoter |  |
| MF9 | 170 | Potri.001G330300 | promoter | DCD (Development and Cell Death) domain protein |
| MF11 | 438 | Potri.001G062100 | 3' flanking region |  |
| MF12 | 333 | Potri.005G172200 | exon | MAPK/ERK kinase kinase 1 |
| MF14 | 496 | Potri.005G114600 | exon | Transducin/WD40 repeat-like superfamily protein |
| MF16 | 134 | Potri.015G003100 | 3' UTR | O-methyltransferase 1 |
| MF18 | 217 | Potri.003G057100 | exon | HXXXD-type acyl-transferase family protein |
| MF19 | 210 | Potri.012G021300 | intron |  |
| MF20 | 175 | Potri.002G078800 | exon | Wound-responsive family protein |
| MF22 | 97 | Potri.014G099300 | exon |  |
| MF23 | 350 | Potri.002G171600 | exon | Uncharacterized conserved protein (DUF2043) |
| MF24 | 95 | Potri.001G424200 | promoter |  |
| MF25 | 208 | Potri.001G424100 | 3' flanking region |  |
| MF26 | 150 | Potri.006G021700 | 3'UTR | Cysteine proteinases superfamily protein |
| MF27 | 154 | Potri.011G022600 | exon | Ubiquitin-specific protease family C19-related protein |
| MF28 | 203 | Potri.010G078600 | promoter | Protein of unknown function (DUF1012) |
| MF30 | 183 | Potri.014G049200 | intron | Glycine-tRNA ligases |
| MF31 | 168 | Potri.006G137600 | exon | Flavin-dependent monooxygenase 1 |
| MF32 | 65 | Potri.008G179600 | 3' flanking region | DNA-binding HORMA family protein |
| MF33 | 338 | Potri.008G179700 | 3' UTR | Homeodomain-like superfamily protein |
| MF35 | 185 | Potri.016G073500 | 3' flanking region |  |
| MF37 | 109 | Potri.013G021400 | intron | Metallopeptidase M24 family protein |
| MF38 | 147 | Potri.017G077400 | promoter | UDP-Glycosyltransferase superfamily protein |
| MF40 | 525 | Potri.014G008600 | exon | VQ motif-containing protein |
| MF41 | 166 | Potri.009G084100 | promoter | AGAMOUS-like 62 |
| MF42 | 289 | Potri.016G047900 | exon | Myb-like HTH transcriptional regulator family protein |
| MF43 | 136 | Potri.010G028100 | promoter | Protein-methionine-S-oxide reductase activity |
| MF45 | 385 | Potri.006G204400 | promoter |  |
| MF46 | 193 | Potri.016G071700 | 5'UTR |  |
| MF48 | 213 | Potri.001G454400 | intron | TMPIT-like protein |
| MF50 | 460 | Potri.009G007800 | exon | ABC transporter family protein |
| MF51 | 276 | Potri.009G008100 | exon | UDP-Glycosyltransferase superfamily protein |
| MF53 | 228 | Potri.016G068900 | intron | RNA helicase family protein |
| MF55 | 331 | Potri.009G014800 | exon | Auxin response factor 10 |
| MF56 | 208 | Potri.009G026600 | 3' flanking region | Hercules receptor kinase 1 |
| MF57 | 119 | Potri.009G026700 | exon | Nudix hydrolase homolog 9 |
| MF58 | 233 | Potri.009G041200 | exon | RNA helicase, ATP-dependent, SK12/DOB1 protein |
| MF59 | 139 | Potri.009G054300 | exon | Origin recognition complex 1 |
| MF60 | 187 | Potri.009G067300 | 3' flanking region | Early nodulin-like protein 18 |
| MF61 | 395 | Potri.009G123100 | intron | Squalene synthase 1 |
| MF62 | 319 | Potri.009G153200 | exon | HSP20-like chaperones superfamily protein |
| MF63 | 527 | Potri.001G399500 | exon | Core-2/I-branching beta-1,6-N-acetylglucosaminyltransferase family protein |
| MF64 | 281 | Potri.001G170300 | 3'UTR | P-loop containing nucleoside triphosphate hydrolases superfamily protein |
| MF65 | 263 | Potri.001G168200 | exon | RELA/SPOT homolog 3 |
| MF66 | 158 | Potri.001G275500 | exon | Non-specific phospholipase C2 |
| MF67 | 423 | Potri.001G344600 | intron | Protein kinase superfamily protein |
| MF69 | 139 | Potri.001G404100 | exon | NAC (No Apical Meristem) domain transcriptional regulator superfamily protein |
| MF70 | 119 | Potri.001G418600 | exon | Translation initiation factor SUI1 family protein |
| MF71 | 151 | Potri.001G129100 | 3' flanking region | Eukaryotic translation initiation factor 2 gamma subunit |
| MF72 | 153 | Potri.001G040500 | exon | Wall associated kinase-like 2 |
| MF73 | 96 | Potri.001G007300 | promoter | Amino acid permease family protein |
| MF75 | 224 | Potri.001G096400 | exon | Cation/H+ exchanger 18 |
| MF76 | 301 | Potri.001G094900 | 5' UTR | Ubiquitin-conjugating enzyme 28 |
| MF77 | 288 | Potri.001G049800 | 3' UTR | Putative type 1 membrane protein |
| MF78 | 163 | Potri.001G157900 | promoter | Peroxin 19-1 |
| MF79 | 210 | Potri.001G161700 | intron | Regulatory particle triple-A 1A |
| MF81 | 492 | Potri.001G171000 | exon | Phospholipid:diacylglycerol acyltransferase |
| MF82 | 342 | Potri.001G188300 | 3' flanking region | Mitotic phosphoprotein N\' end (MPPN) family protein |
| MF84 | 348 | Potri.001G208600 | 3' flanking region | WRKY DNA-binding protein 9 |
| MF88 | 353 | Potri.001G267800 | promoter | ADP/ATP carrier 2 |
| MF89 | 420 | Potri.001G313300 | 3' flanking region | Disease resistance protein (CC-NBS-LRR class) family |
| MF91 | 207 | Potri.001G320300 | exon | DNA repair-recombination protein (RAD50) |
| MF92 | 352 | Potri.001G343500 | intron | Golgin candidate 6 |
| MF93 | 511 | Potri.001G435900 | 3' flanking region | Protein kinase superfamily protein |
| MF94 | 404 | Potri.001G458900 | exon | Peroxidase activity, response to oxidative stress, heme binding, oxidation reduction |
| MF95 | 139 | Potri.001G461000 | promoter | Basic helix-loop-helix (bHLH) DNA-binding superfamily protein |
| MF97 | 389 | Potri.005G229100 | 3' flanking region | PfkB-like carbohydrate kinase family protein |
| MF99 | 144 | Potri.007G123400 | exon | Raffinose synthase family protein |
| MF100 | 235 | Potri.003G175700 | exon | Leucine-rich repeat receptor-like protein kinase family protein |
| MF102 | 205 | Potri.010G098400 | exon | Tetratricopeptide repeat (TPR)-like superfamily protein |
| MF106 | 214 | Potri.005G049100 | exon | Sec23/Sec24 protein transport family protein |
| MF107 | 405 | Potri.005G136000 | promoter | SAP domain-containing protein |
| MF108 | 230 | Potri.005G160900 | 3' flanking region | RING/U-box superfamily protein |
| MF109 | 315 | Potri.001G431800 | 3' flanking region | ENTH/ANTH/VHS superfamily protein |
| MF110 | 363 | Potri.005G140200 | intron | SET domain-containing protein |
| MF111 | 194 | Potri.005G140300 | intron | Plant protein of unknown function (DUF869) |
| MF112 | 389 | Potri.005G179500 | 3' flanking region | Ubiquitin-specific protease 26 |
| MF114 | 368 | Potri.005G251100 | 3' UTR | Small nuclear ribonucleoprotein family protein |
| MF115 | 157 | Potri.016G043900 | exon | Heat shock protein DnaJ with tetratricopeptide repeat |
| MF116 | 205 | Potri.001G314100 | 3' flanking region | Ribosomal protein L31 |
| MF118 | 494 | Potri.005G255400 | intron | Tonoplast monosaccharide transporter2 |
| MF119 | 523 | Potri.005G148300 | exon | Formin homolog 6 |
| MF120 | 213 | Potri.005G148400 | promoter | Integrase-type DNA-binding superfamily protein |
| MF121 | 406 | Potri.003G095400 | 3' UTR | RNA-binding (RRM/RBD/RNP motifs) family protein |
| MF122 | 352 | Potri.007G052600 | 3' flanking region | 2-oxoglutarate (2OG) and Fe(II)-dependent oxygenase superfamily protein |
| MF123 | 205 | Potri.007G066800 | exon | Activator of spomin::LUC2 |
| MF125 | 203 | Potri.001G439400 | intron |  |
| MF128 | 355 | Potri.010G147800 | promoter | Peroxidase superfamily protein |
| MF129 | 307 | Potri.014G013800 | 5' UTR |  |
| MF130 | 241 | Potri.016G119300 | 3' UTR | AMP deaminase, putative / myoadenylate deaminase, putative |
| MF131 | 407 | Potri.016G119400 | exon | UbiA prenyltransferase family protein |
| MF132 | 359 | Potri.017G078100 | promoter | SPFH/Band 7/PHB domain-containing membrane-associated protein family |
| MF133 | 523 | Potri.017G078200 | intron | Voltage-dependent anion channel 1 |
| MF134 | 239 | Potri.019G083000 | intron | ATP-binding cassette family G25 |
| MF135 | 114 | Potri.019G010000 | exon | Chaperone DnaJ-domain superfamily protein |
| MF136 | 181 | Potri.019G005000 | 3' flanking region | Leucine-rich repeat transmembrane protein kinase |
| MF137 | 345 | Potri.001G237600 | promoter | Pathogenesis-related thaumatin superfamily protein |
| MF138 | 105 | Potri.001G162200 | intron | Ubiquitin-conjugating enzyme 33 |
| MF139 | 184 | Potri.013G069900 | intron | Seven transmembrane MLO family protein |
| MF140 | 144 | Potri.015G132800 | exon | Cytochrome P450, family 96, subfamily A, polypeptide 10 |
| MF141 | 195 | Potri.008G026100 | exon | Cysteine proteinases superfamily protein |
| MF142 | 182 | Potri.002G192800 | 3' flanking region | Zinc knuckle (CCHC-type) family protein |
| MF143 | 122 | Potri.002G105000 | promoter | Predicted AT-hook DNA-binding family protein |
| MF144 | 284 | Potri.002G105100 | 3' flanking region | Clathrin adaptor complexes medium subunit family protein |
| MF145 | 232 | Potri.003G023000 | promoter | BRI1-associated receptor kinase |
| MF146 | 425 | Potri.003G023300 | 3' flanking region | Loricrin-related |
| MF149 | 201 | Potri.002G034700 | exon | Oxidoreductases, acting on NADH or NADPH |
| MF150 | 412 | Potri.004G177600 | exon | Protein of unknown function (DUF616) |
| MF151 | 327 | Potri.004G177700 | exon | Transportin 1 |
| MF152 | 455 | Potri.001G405400 | exon |  |
| MF153 | 365 | Potri.005G058300 | promoter | Callose synthase 5 |
| MF154 | 261 | Potri.007G097100 | promoter | Homeobox protein 16 |
| MF155 | 202 | Potri.010G014600 | promoter | Protein arginine methyltransferase 4A |
| MF156 | 216 | Potri.010G235800 | exon | Pleiotropic drug resistance 6 |
| MF157 | 247 | Potri.010G235900 | exon | Enhancer of polycomb-like transcription factor protein |
| MF158 | 320 | Potri.012G068800 | exon | Pyridine nucleotide-disulphide oxidoreductase family protein |
| MF159 | 272 | Potri.014G009000 | exon | CYCLIN D1;1 |
| MF160 | 383 | Potri.016G066600 | 3' UTR | TPX2 (targeting protein for Xklp2) protein family |
| MF162 | 319 | Potri.005G036400 | intron | RING/U-box superfamily protein |
| MF163 | 312 | Potri.015G082400 | exon | Tetratricopeptide repeat (TPR)-like superfamily protein |
| MF164 | 390 | Potri.003G192400 | exon | Microtubule-associated protein 65-5 |
| MF165 | 511 | Potri.006G072300 | promoter |  |
| MF166 | 243 | Potri.009G058200 | exon | Polyamine oxidase 1 |
| MF167 | 370 | Potri.009G058400 | exon | Tetratricopeptide repeat (TPR)-like superfamily protein |
| MF168 | 155 | Potri.007G033400 | exon |  |
| MF169 | 168 | Potri.003G124100 | exon | DEA(D/H)-box RNA helicase family protein |
| MF170 | 268 | Potri.010G184500 | intron | Ferritin 2 |
| MF172 | 200 | Potri.006G255100 | intron | Leucine-rich repeat transmembrane protein kinase |
| MF178 | 186 | Potri.019G073200 | promoter | Hydroxysteroid dehydrogenase 5 |
| MF179 | 188 | Potri.013G100200 | exon | Oxidoreductase activity |
| MF180 | 370 | Potri.006G124800 | promoter | Heavy metal transport/detoxification superfamily protein |
| MF181 | 178 | Potri.010G069800 | exon | Cysteine-rich RLK (RECEPTOR-like protein kinase) 29 |
| MF182 | 514 | Potri.011G049700 | exon | Sensitive to freezing 6 |
| MF183 | 264 | Potri.016G023000 | 3' flanking region | Membrane trafficking VPS53 family protein |
| MF184 | 346 | Potri.004G087600 | exon | Protein of unknown function, DUF593 |
| MF186 | 411 | Potri.009G032000 | promoter | TRF-like 2 |
| MF187 | 115 | Potri.009G032100 | intron | Rab5-interacting family protein |
| MF188 | 454 | Potri.006G136400 | intron | Phosphoinositide phosphatase family protein |
| MF189 | 262 | Potri.019G076200 | exon | queuine tRNA-ribosyltransferase activity |
| MF190 | 252 | Potri.013G154900 | 3' flanking region | PAP/OAS1 substrate-binding domain superfamily |
| MF191 | 279 | Potri.013G155100 | exon | With no lysine (K) kinase 4 |
| MF192 | 245 | Potri.004G163600 | 3' flanking region | 3-oxo-5-alpha-steroid 4-dehydrogenase family protein |
| MF193 | 474 | Potri.008G069500 | intron | Phosphoribosylanthranilate isomerase 1 |
| MF194 | 448 | Potri.008G070600 | promoter | Clathrin, heavy chain |
| MF195 | 359 | Potri.012G145600 | intron | Phosphoribosylanthranilate isomerase 1 |
| MF196 | 203 | Potri.005G206000 | exon | TRAM, LAG1 and CLN8 (TLC) lipid-sensing domain containing protein |
| MF198 | 223 | Potri.010G081400 | exon | Leucine-rich repeat protein kinase family protein |
| MF199 | 178 | Potri.008G032700 | intron | Pectin lyase-like superfamily protein |
| MF200 | 286 | Potri.006G074700 | 3' flanking region | Basic helix-loop-helix (bHLH) DNA-binding superfamily protein |
| MF202 | 200 | Potri.011G082500 | exon | RNA-binding KH domain-containing protein |
| MF203 | 464 | Potri.012G076500 | exon | Peroxidase superfamily protein |
| MF207 | 347 | Potri.003G121800 | intron | SEUSS-like 2 |
| MF208 | 332 | Potri.003G078000 | exon | Formyltetrahydrofolate deformylase, putative |
| MF209 | 270 | Potri.004G155000 | intron | Phenylalanyl-tRNA synthetase, putative / phenylalanine--tRNA ligase, putative |
| MF210 | 187 | Potri.011G008600 | exon | Transmembrane receptors; ATP binding |
| MF211 | 346 | Potri.001G070300 | intron | C-8,7 sterol isomerase |
| MF212 | 316 | Potri.017G113800 | promoter |  |
| MF213 | 307 | Potri.010G212500 | exon | Transferase activity, fatty acid biosynthetic process |
| MF214 | 341 | Potri.014G002200 | promoter | LRR and NB-ARC domains-containing disease resistance protein |
| MF215 | 549 | Potri.007G140400 | promoter | BTB and TAZ domain protein 3 |
| MF216 | 300 | Potri.001G211300 | exon | Pentatricopeptide repeat (PPR) superfamily protein |
| MF217 | 225 | Potri.003G186300 | exon | Polynucleotidyl transferase, ribonuclease H-like superfamily protein |
| MF219 | 287 | Potri.003G027400 | 3' UTR |  |
| MF220 | 127 | Potri.003G012400 | 3' UTR |  |
| MF221 | 359 | Potri.001G053000 | exon |  |
| MF222 | 169 | Potri.001G103300 | 3' UTR | Acid-amino acid ligases; ligases; ATP binding; ATP binding; ligases |
| MF223 | 319 | Potri.001G180200 | exon | RAD3-like DNA-binding helicase protein |
| MF224 | 224 | Potri.001G401300 | exon | Laccase 17 |
| MF225 | 280 | Potri.004G024500 | intron | Cysteine-rich RLK (RECEPTOR-like protein kinase) 10 |
| MF226 | 328 | Potri.009G078400 | exon | UDP-glucosyl transferase 78D2 |
| MF227 | 462 | Potri.005G146400 | exon | Phototropic-responsive NPH3 family protein |
| MF228 | 199 | Potri.005G040600 | 3' UTR | alpha/beta-Hydrolases superfamily protein |
| MF229 | 297 | Potri.005G079400 | promoter | Cupredoxin superfamily protein |
| MF230 | 289 | Potri.017G114800 | intron | alpha/beta-Hydrolases superfamily protein |
| MF231 | 351 | Potri.014G015100 | exon | Xanthine/uracil permease family protein |
| MF232 | 352 | Potri.017G063900 | 3' flanking region | Ferritin/ribonucleotide reductase-like family protein |
| MF233 | 383 | Potri.002G156200 | intron | Calcineurin-like metallo-phosphoesterase superfamily protein |
| MF234 | 520 | Potri.016G006500 | exon | Oligopeptide transporter |
| MF235 | 368 | Potri.010G018800 | exon | DNAJ heat shock N-terminal domain-containing protein |
| MF236 | 327 | Potri.010G029100 | intron | Glutamine synthetase 2 |
| MF237 | 321 | Potri.010G034300 | 3'UTR | Nitrate transporter 1:2 |
| MF238 | 139 | Potri.010G106200 | promoter | Cleavage and polyadenylation specificity factor 73 kDa subunit-II |
| MF239 | 357 | Potri.010G114400 | exon | AICARFT/IMPCHase bienzyme family protein |
| MF240 | 148 | Potri.010G116900 | promoter | MATE efflux family protein |
| MF241 | 320 | Potri.010G119300 | promoter | Lipid phosphate phosphatase 2 |
