# Supplementary material for: The Role of DNA Methylation in Xylogenesis in Different Tissues of Poplar
Source: Front Plant Sci. 2016 Jul 12;7:1003. doi: 10.3389/fpls.2016.01003 (PMC4941658; doi:10.3389/fpls.2016.01003)
Supplement: Supplementary file 6 [file Table6.DOC]

**Table S6.** Differential cytosine methylation patterns of 210 polymorphic MSAP fragments in tissues and organs

| MSAP  fragment | Cytosine methylation patterns | | | | | | | | | | Methylation  specificity |
| --- | --- | --- | --- | --- | --- | --- | --- | --- | --- | --- | --- |
|  | Young  leaf | Mature  leaf | Phloem | Cambium | Root | Shoot  apex | Developing  xylem | Mature  xylem | Male  catkin | Female  catkin |  |
| MSAP fragment1 | F | U | F | F | F | F | F | F | U | U | 0.667 |
| MSAP fragment2 | F | U | F | F | U | F | F | F | F | F | 0.778 |
| MSAP fragment4 | F | N | F | N | F | N | F | F | F | U | 0.556 |
| MSAP fragment6 | U | F | F | N | F | F | F | F | F | U | 0.667 |
| MSAP fragment7 | U | U | F | N | N | N | F | U | U | U | 0.111 |
| MSAP fragment9 | F | H | N | N | F | N | F | F | F | F | 0.667 |
| MSAP fragment11 | U | F | N | N | F | N | U | U | U | U | 0.111 |
| MSAP fragment12 | F | N | N | N | F | N | U | U | U | U | 0.111 |
| MSAP fragment14 | F | U | N | N | F | N | N | H | N | F | 0.333 |
| MSAP fragment16 | F | F | N | F | F | N | U | U | U | F | 0.444 |
| MSAP fragment17 | H | N | N | N | F | N | N | F | N | F | 0.333 |
| MSAP fragment18 | U | H | N | F | U | F | H | F | N | F | 0.556 |
| MSAP fragment19 | U | H | N | N | F | F | H | F | N | F | 0.556 |
| MSAP fragment20 | U | H | N | N | F | N | H | F | N | U | 0.333 |
| MSAP fragment21 | U | H | N | N | N | F | N | F | N | F | 0.333 |
| MSAP fragment22 | U | U | U | F | U | H | F | U | F | F | 0.444 |
| MSAP fragment23 | U | H | H | N | U | F | U | U | N | F | 0.333 |
| MSAP fragment24 | F | F | F | F | U | F | F | F | U | U | 0.667 |
| MSAP fragment25 | H | N | N | N | F | N | F | H | U | U | 0.333 |
| MSAP fragment26 | U | N | F | H | F | N | U | N | U | U | 0.222 |
| MSAP fragment27 | U | H | H | H | H | N | H | F | N | U | 0.556 |
| MSAP fragment28 | U | U | F | F | F | N | U | U | F | U | 0.333 |
| MSAP fragment30 | U | H | U | N | N | N | U | H | F | U | 0.222 |
| MSAP fragment31 | U | N | N | N | F | N | F | F | U | U | 0.222 |
| MSAP fragment32 | F | N | H | N | N | N | F | F | F | U | 0.444 |
| MSAP fragment33 | U | N | N | N | N | N | N | U | U | H | 0.000 |
| MSAP fragment35 | H | H | H | N | U | N | U | F | U | U | 0.333 |
| MSAP fragment36 | U | H | H | N | U | N | U | U | U | U | 0.111 |
| MSAP fragment37 | H | H | H | N | U | N | N | U | U | H | 0.333 |
| MSAP fragment38 | U | U | H | N | U | N | H | U | F | U | 0.222 |
| MSAP fragment40 | U | F | U | N | U | H | N | U | F | U | 0.222 |
| MSAP fragment41 | H | H | H | H | U | H | H | U | U | U | 0.556 |
| MSAP fragment42 | F | F | F | F | U | F | F | F | N | U | 0.667 |
| MSAP fragment43 | U | U | H | N | H | N | H | H | F | U | 0.444 |
| MSAP fragment44 | U | H | H | N | H | N | H | U | F | U | 0.444 |
| MSAP fragment45 | N | U | N | N | U | N | N | U | F | U | 0.000 |
| MSAP fragment46 | H | H | H | U | U | N | U | U | F | U | 0.333 |
| MSAP fragment48 | H | H | F | N | U | N | N | F | N | H | 0.444 |
| MSAP fragment50 | H | H | N | N | U | N | N | U | N | U | 0.111 |
| MSAP fragment51 | H | H | N | N | U | N | U | F | N | U | 0.222 |
| MSAP fragment53 | H | N | N | N | U | N | F | F | N | U | 0.222 |
| MSAP fragment55 | H | H | H | N | U | N | F | U | N | U | 0.333 |
| MSAP fragment56 | U | U | H | N | U | N | F | U | N | U | 0.111 |
| MSAP fragment57 | H | H | N | N | U | F | F | F | F | F | 0.667 |
| MSAP fragment58 | H | F | F | F | F | F | F | F | F | H | 1.000 |
| MSAP fragment59 | U | F | F | F | F | N | F | F | N | U | 0.556 |
| MSAP fragment60 | U | F | F | F | F | F | F | F | F | U | 0.778 |
| MSAP fragment61 | U | F | F | F | F | F | F | F | F | U | 0.778 |
| MSAP fragment62 | U | F | F | F | F | F | F | F | F | U | 0.778 |
| MSAP fragment63 | U | F | F | F | F | F | F | F | F | F | 0.889 |
| MSAP fragment64 | U | F | F | F | F | F | F | F | N | N | 0.667 |
| MSAP fragment65 | U | N | F | F | F | F | F | F | U | U | 0.556 |
| MSAP fragment66 | U | N | F | F | F | F | F | F | U | N | 0.556 |
| MSAP fragment67 | F | F | F | F | F | F | F | F | N | U | 0.778 |
| MSAP fragment69 | U | F | F | F | F | F | F | F | F | U | 0.778 |
| MSAP fragment70 | F | N | F | F | F | N | F | F | N | U | 0.556 |
| MSAP fragment71 | F | N | F | F | F | F | F | F | N | N | 0.667 |
| MSAP fragment72 | F | N | F | F | F | F | F | F | N | N | 0.667 |
| MSAP fragment73 | U | F | F | F | F | F | F | F | U | U | 0.667 |
| MSAP fragment75 | F | F | N | F | N | N | N | N | N | H | 0.333 |
| MSAP fragment76 | F | F | N | H | N | N | N | H | N | H | 0.444 |
| MSAP fragment77 | F | N | U | N | F | F | U | H | H | U | 0.444 |
| MSAP fragment78 | F | N | H | N | F | F | H | H | N | N | 0.556 |
| MSAP fragment79 | N | N | H | N | F | N | H | H | U | U | 0.333 |
| MSAP fragment81 | U | F | U | N | F | F | H | U | F | U | 0.444 |
| MSAP fragment82 | U | H | U | H | U | H | H | H | U | U | 0.444 |
| MSAP fragment84 | F | N | U | N | F | N | H | H | F | U | 0.444 |
| MSAP fragment88 | U | F | F | N | F | F | H | U | F | U | 0.556 |
| MSAP fragment89 | U | H | F | N | U | F | N | H | F | H | 0.556 |
| MSAP fragment91 | U | H | F | N | U | F | F | U | F | H | 0.556 |
| MSAP fragment92 | U | H | F | N | U | F | F | F | U | U | 0.444 |
| MSAP fragment93 | U | H | F | F | U | U | F | F | U | U | 0.444 |
| MSAP fragment94 | U | H | F | N | U | U | F | F | U | H | 0.444 |
| MSAP fragment95 | U | H | F | N | U | F | N | F | U | H | 0.444 |
| MSAP fragment96 | F | F | F | F | U | N | N | U | N | N | 0.333 |
| MSAP fragment97 | F | F | U | F | U | N | N | U | N | N | 0.222 |
| MSAP fragment98 | U | F | U | F | U | F | H | U | H | F | 0.556 |
| MSAP fragment99 | F | F | U | F | U | F | H | U | H | F | 0.667 |
| MSAP fragment100 | F | F | U | F | U | F | H | U | H | N | 0.556 |
| MSAP fragment102 | U | H | N | N | H | F | H | H | H | F | 0.667 |
| MSAP fragment106 | U | N | F | F | U | N | N | F | N | F | 0.333 |
| MSAP fragment107 | F | F | F | H | N | F | H | H | N | N | 0.667 |
| MSAP fragment108 | U | N | F | N | F | N | F | F | F | U | 0.444 |
| MSAP fragment109 | F | H | N | N | F | N | F | F | F | F | 0.667 |
| MSAP fragment110 | F | N | F | F | F | F | F | F | N | F | 0.778 |
| MSAP fragment111 | F | F | F | F | F | F | F | F | N | F | 0.889 |
| MSAP fragment112 | N | N | H | U | U | U | N | F | F | U | 0.222 |
| MSAP fragment114 | N | U | N | N | N | N | N | F | N | U | 0.000 |
| MSAP fragment115 | N | U | N | N | U | N | N | H | H | H | 0.222 |
| MSAP fragment116 | N | N | N | N | F | N | N | N | U | H | 0.111 |
| MSAP fragment117 | N | N | N | F | F | F | F | F | F | U | 0.556 |
| MSAP fragment118 | N | N | N | N | F | F | F | F | F | U | 0.444 |
| MSAP fragment119 | N | N | F | N | F | N | F | F | N | U | 0.333 |
| MSAP fragment120 | F | F | F | F | F | F | F | F | U | U | 0.778 |
| MSAP fragment121 | H | H | H | H | N | U | H | H | U | U | 0.556 |
| MSAP fragment122 | H | H | U | U | H | U | H | H | U | H | 0.556 |
| MSAP fragment123 | U | U | N | F | U | N | F | U | N | U | 0.111 |
| MSAP fragment125 | U | F | N | N | F | N | U | U | U | U | 0.111 |
| MSAP fragment128 | F | N | N | N | F | N | U | F | H | U | 0.333 |
| MSAP fragment129 | N | F | N | N | U | N | N | U | N | N | 0.000 |
| MSAP fragment130 | F | F | F | F | F | F | F | F | N | F | 0.889 |
| MSAP fragment131 | F | F | F | F | F | F | F | F | N | F | 0.889 |
| MSAP fragment132 | U | N | U | U | U | U | N | U | N | F | 0.000 |
| MSAP fragment133 | H | N | N | H | H | H | N | F | N | F | 0.556 |
| MSAP fragment134 | U | U | N | N | U | N | F | F | F | U | 0.222 |
| MSAP fragment135 | N | N | N | N | U | N | F | U | N | N | 0.000 |
| MSAP fragment136 | F | N | N | N | N | N | H | H | N | U | 0.222 |
| MSAP fragment137 | F | F | N | N | N | H | N | U | U | H | 0.333 |
| MSAP fragment138 | H | N | H | H | H | H | U | U | N | F | 0.556 |
| MSAP fragment139 | U | N | N | N | H | N | F | U | F | U | 0.222 |
| MSAP fragment140 | F | F | F | F | F | F | F | F | F | N | 0.889 |
| MSAP fragment141 | U | N | F | F | U | N | F | F | F | U | 0.444 |
| MSAP fragment142 | H | H | N | N | U | N | N | U | N | U | 0.111 |
| MSAP fragment143 | H | H | F | N | U | N | F | F | N | U | 0.444 |
| MSAP fragment144 | H | U | N | N | N | U | U | U | U | N | 0.000 |
| MSAP fragment145 | U | U | H | N | N | U | U | U | N | F | 0.111 |
| MSAP fragment146 | F | F | F | F | F | F | F | F | F | N | 0.889 |
| MSAP fragment147 | F | F | F | F | U | F | F | F | N | U | 0.667 |
| MSAP fragment148 | F | F | F | F | F | F | F | F | N | U | 0.778 |
| MSAP fragment149 | F | F | F | N | N | N | N | N | U | F | 0.333 |
| MSAP fragment150 | N | N | F | F | U | F | F | F | F | U | 0.556 |
| MSAP fragment151 | N | N | N | N | N | N | N | N | U | H | 0.000 |
| MSAP fragment152 | F | N | F | F | F | F | F | F | N | N | 0.667 |
| MSAP fragment153 | N | H | H | H | H | H | U | U | H | H | 0.667 |
| MSAP fragment154 | N | F | N | F | F | F | F | F | F | U | 0.667 |
| MSAP fragment155 | N | H | F | F | F | N | N | F | U | F | 0.556 |
| MSAP fragment156 | N | N | N | N | N | H | H | H | U | H | 0.333 |
| MSAP fragment157 | N | N | N | N | H | H | H | H | H | H | 0.556 |
| MSAP fragment158 | N | N | N | N | N | H | H | H | H | H | 0.444 |
| MSAP fragment159 | H | N | N | N | F | H | H | H | H | H | 0.667 |
| MSAP fragment160 | H | N | N | N | H | H | H | H | H | H | 0.667 |
| MSAP fragment161 | H | N | N | H | H | H | H | U | H | U | 0.556 |
| MSAP fragment162 | U | U | U | U | U | U | H | H | U | F | 0.222 |
| MSAP fragment163 | N | F | F | F | F | U | N | F | N | F | 0.556 |
| MSAP fragment164 | U | U | U | U | H | U | H | H | U | U | 0.222 |
| MSAP fragment165 | F | U | F | N | H | N | N | U | N | N | 0.222 |
| MSAP fragment166 | F | F | F | F | F | F | F | F | F | U | 0.889 |
| MSAP fragment167 | F | H | H | H | N | H | H | H | N | F | 0.778 |
| MSAP fragment168 | F | N | N | N | F | F | H | H | F | N | 0.556 |
| MSAP fragment169 | F | U | F | F | F | N | F | F | U | N | 0.556 |
| MSAP fragment170 | F | U | F | F | F | N | F | F | U | N | 0.556 |
| MSAP fragment171 | N | H | H | H | H | N | H | H | N | N | 0.556 |
| MSAP fragment172 | F | F | U | H | N | F | F | F | U | U | 0.556 |
| MSAP fragment173 | N | F | F | N | N | N | N | N | F | H | 0.333 |
| MSAP fragment174 | F | N | N | N | F | N | H | H | H | N | 0.444 |
| MSAP fragment175 | H | N | N | N | U | U | U | U | N | N | 0.000 |
| MSAP fragment176 | H | N | H | N | H | H | H | H | N | U | 0.556 |
| MSAP fragment178 | H | N | N | N | N | N | N | F | N | U | 0.111 |
| MSAP fragment179 | F | F | F | F | F | F | F | F | U | U | 0.778 |
| MSAP fragment180 | F | N | F | N | F | F | H | H | F | N | 0.667 |
| MSAP fragment181 | N | U | H | H | N | H | F | N | U | U | 0.333 |
| MSAP fragment182 | N | H | H | H | U | H | H | H | H | H | 0.778 |
| MSAP fragment183 | H | N | N | N | U | H | U | F | N | U | 0.222 |
| MSAP fragment184 | H | N | N | N | N | N | N | N | N | N | 0.000 |
| MSAP fragment186 | F | F | F | F | F | F | F | F | N | N | 0.778 |
| MSAP fragment187 | F | F | F | F | F | F | F | F | N | N | 0.778 |
| MSAP fragment188 | N | N | F | F | F | F | F | F | F | F | 0.778 |
| MSAP fragment189 | N | N | F | N | H | U | U | N | F | U | 0.222 |
| MSAP fragment190 | F | F | U | U | U | U | U | U | U | U | 0.111 |
| MSAP fragment191 | N | H | N | N | H | H | N | N | N | N | 0.222 |
| MSAP fragment192 | N | H | F | N | N | F | N | H | F | N | 0.444 |
| MSAP fragment193 | N | N | F | N | N | F | U | N | U | U | 0.111 |
| MSAP fragment194 | U | F | N | F | H | F | H | N | H | F | 0.667 |
| MSAP fragment195 | F | F | N | F | N | F | N | H | F | H | 0.667 |
| MSAP fragment196 | H | H | H | H | U | H | H | N | U | N | 0.556 |
| MSAP fragment197 | H | N | N | H | H | H | H | N | U | H | 0.556 |
| MSAP fragment198 | F | H | F | F | H | F | N | H | F | H | 0.889 |
| MSAP fragment199 | H | H | F | F | N | F | N | H | F | N | 0.667 |
| MSAP fragment200 | N | N | N | N | N | N | H | H | H | H | 0.333 |
| MSAP fragment201 | N | N | N | N | N | N | N | N | H | H | 0.111 |
| MSAP fragment202 | N | N | N | N | N | N | N | N | H | H | 0.111 |
| MSAP fragment203 | H | N | N | H | H | N | H | H | H | H | 0.667 |
| MSAP fragment204 | H | N | H | N | H | N | N | H | H | H | 0.556 |
| MSAP fragment205 | N | F | U | F | N | F | N | N | H | H | 0.444 |
| MSAP fragment206 | N | U | U | F | N | F | N | N | H | H | 0.333 |
| MSAP fragment207 | F | F | F | U | F | F | F | F | U | U | 0.667 |
| MSAP fragment208 | U | N | N | H | U | N | F | F | U | U | 0.222 |
| MSAP fragment209 | U | N | N | N | U | N | F | F | U | U | 0.111 |
| MSAP fragment210 | U | N | N | H | H | N | U | F | N | U | 0.222 |
| MSAP fragment211 | N | N | N | H | N | N | N | F | N | N | 0.111 |
| MSAP fragment212 | N | N | F | F | F | N | F | F | N | F | 0.556 |
| MSAP fragment213 | N | U | H | H | H | N | N | F | U | N | 0.333 |
| MSAP fragment214 | N | H | N | N | H | N | N | F | N | N | 0.222 |
| MSAP fragment215 | N | F | F | F | F | F | N | F | F | F | 0.778 |
| MSAP fragment216 | N | N | F | F | F | U | F | F | F | U | 0.556 |
| MSAP fragment217 | H | H | H | H | N | H | H | U | U | U | 0.556 |
| MSAP fragment218 | N | N | N | N | N | H | N | U | U | U | 0.000 |
| MSAP fragment219 | U | N | F | F | F | U | U | U | U | U | 0.222 |
| MSAP fragment220 | N | H | H | N | N | N | N | N | U | N | 0.111 |
| MSAP fragment221 | F | F | N | F | U | N | F | N | N | F | 0.444 |
| MSAP fragment222 | F | F | F | F | F | F | F | F | N | F | 0.889 |
| MSAP fragment223 | H | H | H | H | H | H | H | H | N | N | 0.778 |
| MSAP fragment224 | H | N | H | N | H | H | H | H | N | N | 0.556 |
| MSAP fragment225 | F | F | F | F | F | F | F | F | F | N | 0.889 |
| MSAP fragment226 | N | N | H | F | F | F | H | U | N | N | 0.444 |
| MSAP fragment227 | U | N | N | N | H | N | F | U | F | F | 0.333 |
| MSAP fragment228 | F | N | F | F | U | N | F | F | F | H | 0.667 |
| MSAP fragment229 | H | H | N | N | U | N | N | N | N | H | 0.222 |
| MSAP fragment230 | H | H | F | N | H | N | F | F | N | H | 0.667 |
| MSAP fragment231 | F | F | F | F | H | F | F | F | N | U | 0.778 |
| MSAP fragment232 | H | H | N | N | U | F | F | F | F | F | 0.667 |
| MSAP fragment233 | H | F | F | F | F | F | F | F | F | H | 1.000 |
| MSAP fragment234 | U | F | F | F | F | N | F | F | N | U | 0.556 |
| MSAP fragment235 | U | U | F | F | F | F | F | F | H | F | 0.778 |
| MSAP fragment236 | F | N | U | N | F | F | H | H | F | U | 0.556 |
| MSAP fragment237 | F | N | U | N | F | N | H | H | H | U | 0.444 |
| MSAP fragment238 | F | N | F | N | F | F | H | H | F | U | 0.667 |
| MSAP fragment239 | U | F | U | F | N | F | H | H | H | F | 0.667 |
| MSAP fragment240 | N | H | N | N | N | N | N | F | N | H | 0.222 |
| MSAP fragment241 | H | N | H | N | H | F | H | H | N | H | 0.667 |

H, F, U, and N indicated hemi-methylation, full methylation, uninformative site, and non-methylation, respectively.
