# Supplementary material for: The Role of DNA Methylation in Xylogenesis in Different Tissues of Poplar
Source: Front Plant Sci. 2016 Jul 12;7:1003. doi: 10.3389/fpls.2016.01003 (PMC4941658; doi:10.3389/fpls.2016.01003)
Supplement: Supplementary file 8 [file Image1.PDF]

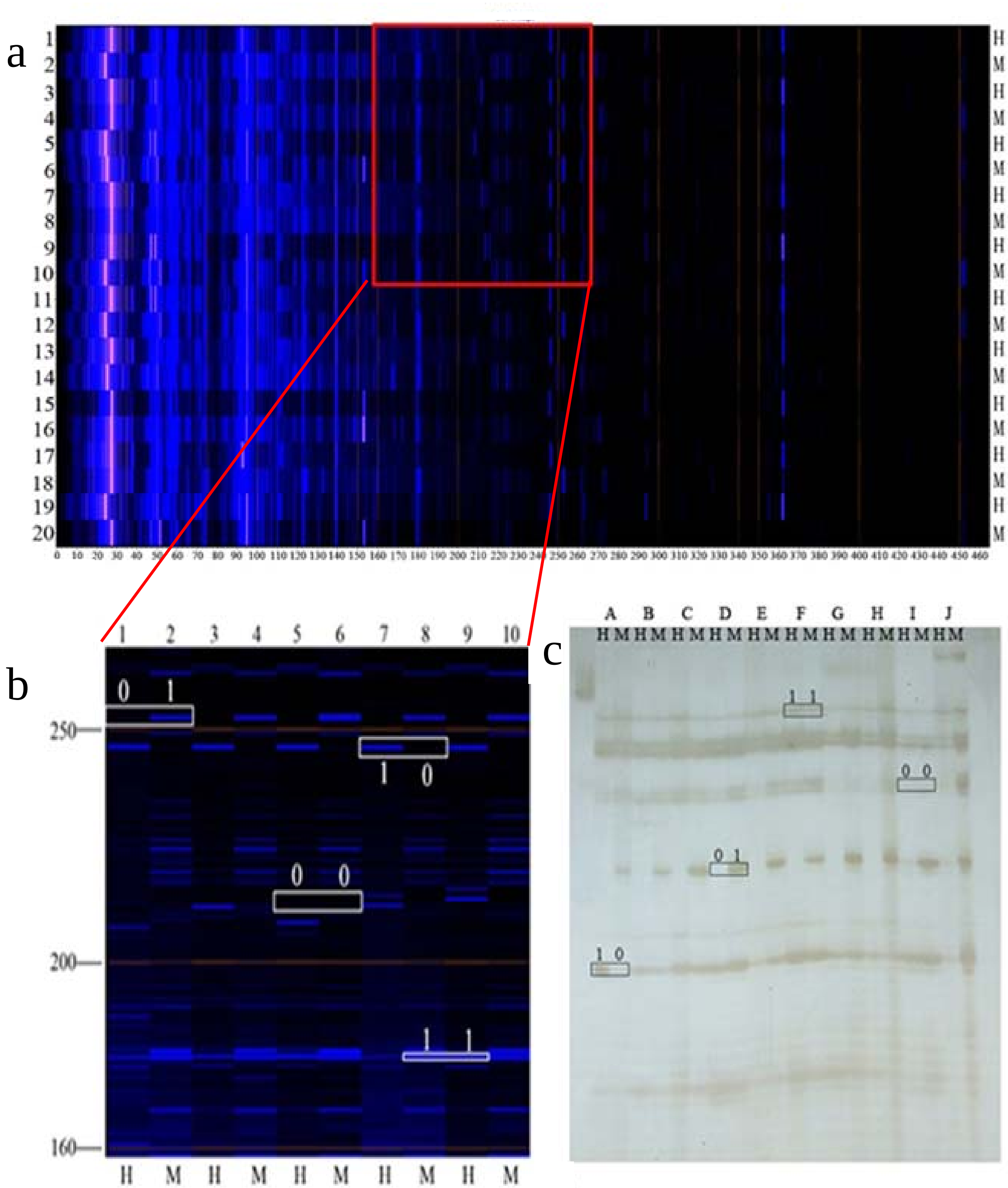

Fig. S1 Methylation-sensitive amplified polymorphism (MSAP) analysis of ten tissue and organ types of *P. tomentosa*. The primer-pair combination E66+H/M33 was used for amplification. H and M represent samples from ten tissue and organ types, where the genomic DNAs were digested with *Eco*R I/*Hpa*II, and *Eco*R I/*Msp*I, respectively. (1, 0), (0, 1), (0, 0), and (1, 1) indicated hemi-methylation, full methylation, uninformative site, and

non-methylation, respectively. (a) Gel image generated by GeneMarker V2.2.0 (Softgenetics, State College, PA, USA) software after selective amplification products were separated by capillary electrophoresis and visualized by fluorescent detection. Blue bands show amplification products and orange bands show standard samples. The numbers on the left indicate lane numbers, every two lanes were one tissue or organ type, and the numbers below represent fragment length (bp). (b) Enlargement of one part of (a) in the red frame, rotated 90° counter clockwise, displaying four patterns of band combinations. (c) Electrophoretic bands of amplified fragments on a 6% polyacrylamide sequencing gel. A-J represent young leaf, mature leaf, phloem, cambium, root, shoot apex, developing xylem, mature xylem, male catkin, and female catkin, respectively.
