# Supplementary material for: The Role of DNA Methylation in Xylogenesis in Different Tissues of Poplar
Source: Front Plant Sci. 2016 Jul 12;7:1003. doi: 10.3389/fpls.2016.01003 (PMC4941658; doi:10.3389/fpls.2016.01003)
Supplement: Supplementary file 9 [file Image2.PDF]

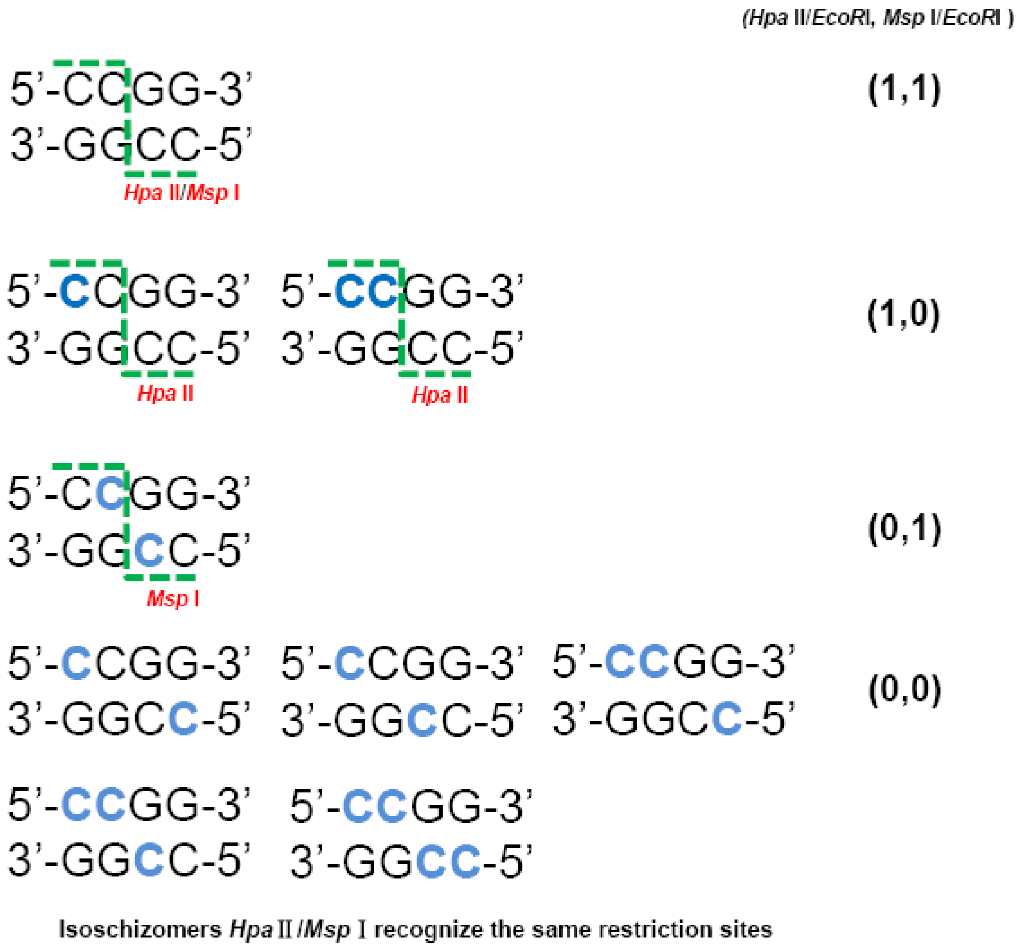

Figure S2. Molecular basis of MSAP. Isoschizomers *Hpa* II /*Msp* I recognize the same restriction 5'-CCGG site but have different sensitivety to methylation of the cytosines. *Hpa* II digests if the external cytosine is hemimethylated (single-strand) or non-methyled 5'-CCGG site but does not digest if either of the cytosines is fully (double-strand) methylated, while *Msp* I does not, cutting only if the internal cytosine is fully (double-strand) methylated or specifically non-methyled 5'-CCGG site. Neither *Hpa* II nor *Msp* I digests methylation that happened on four cytosines of a 5'-CCGG site (doublestrand) at the same time or some other combinations. Blue represent the methylated cytosines. Green line represents methylation sensitive restriction enzyme digestion. '1'and '0' represent statistics of electrophoretic band. For clarity, (1, 0), (0, 1), (0, 0), and (1, 1) indicated hemi-methylation, full methylation, uninformative site, and non-methylation, respectively. Adapted from Ci et al. (2015).
