# Supplementary material for: The Role of DNA Methylation in Xylogenesis in Different Tissues of Poplar
Source: Front Plant Sci. 2016 Jul 12;7:1003. doi: 10.3389/fpls.2016.01003 (PMC4941658; doi:10.3389/fpls.2016.01003)
Supplement: Supplementary file 10 [file Image3.PDF]

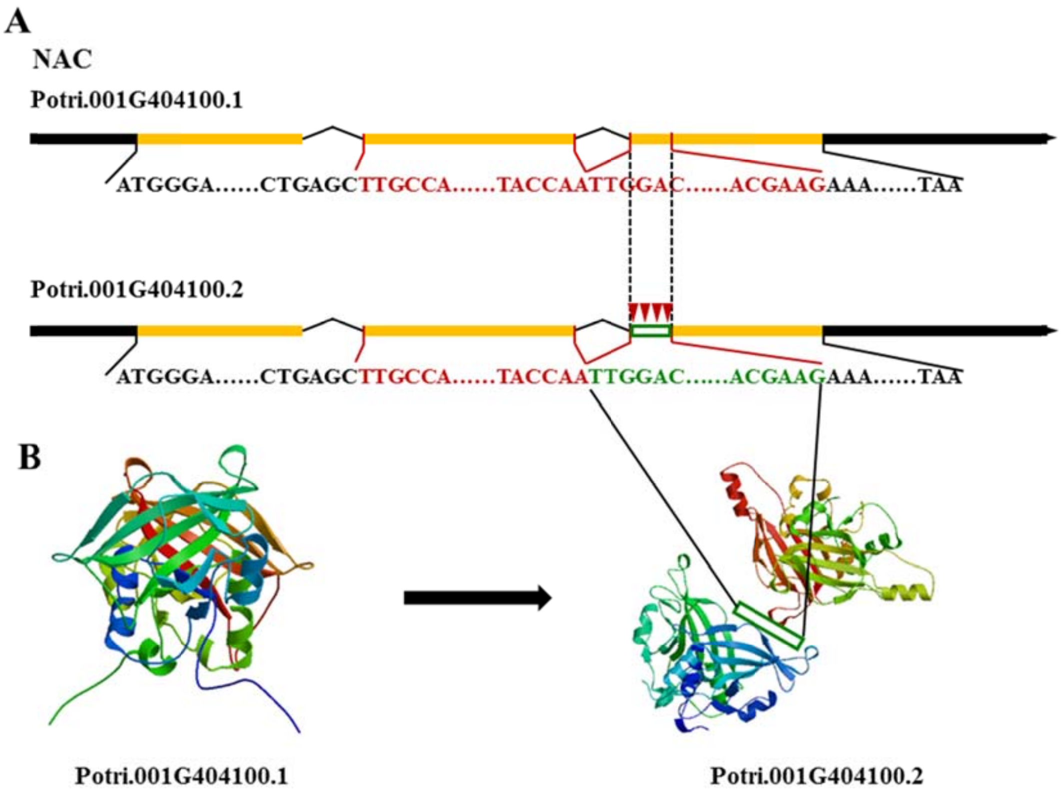

Fig. S3 Different transcripts of *NAC* encoding different protein. (A) , red inverted triangle, represents methyl and red words represent DNA encoding the functional domain where green words represent DNA encoding the key domain. (B) The two different 3D-structure of NAC are translated by different transcripts, respectively. ,
green rectangle, represents the lost part of the third exon in the second transcript.
